# A star-shaped triazine-BODIPY trimer with fluorene spacers: synthesis, aggregation, and environment-dependent photophysical properties

**DOI:** 10.1039/d6ra05235e

**Published:** 2026-09-21

**Authors:** Laxmipriya Nayak, Harshal V. Barkale, Subhadeep Acharya, Supriya Routray, Simran Pattnaik, Nilanjan Dey, Rashmirekha Satapathy

**Affiliations:** a Department of Chemistry, Ravenshaw University Cuttack-753003 Odisha India rashmi@ravenshawuniversity.ac.in; b Department of Chemistry, Birla Institute of Technology and Science, Pilani Hyderabad-500078 Telangana India nilanjan@hyderabad.bits-pilani.ac.in

## Abstract

A novel star-shaped triazine-BODIPY trimer incorporating fluorene spacers (Tz-Fl-BDP) was successfully synthesized and well characterized. Experimental and computational investigations were carried out to elucidate its structural, electronic, and excited-state properties. The photophysical behavior of Tz-Fl-BDP was examined across solvents of varying polarity using absorption, steady-state, and time-resolved fluorescence, and dynamic light scattering (DLS) analyses. The molecule exhibited two dominant absorption bands at ∼417 and 530 nm, corresponding to higher-energy donor-to-acceptor charge-transfer transitions (ICT) and lower-energy localized BODIPY-centered π → π* transitions, respectively. Strong emission was observed in nonpolar solvents but progressively quenched in polar protic media owing to solvent-promoted non-radiative decay, while red-shifted, broadened emission in water is consistent with aggregation-induced quenching (AIQ). Time-resolved fluorescence and DLS studies correlated solvent-dependent aggregation with excited-state relaxation dynamics. A weak fluorescence quenching (1.47-fold) was observed upon addition of triethylamine (final concentration ≈ 1.25 mM) in THF, suggesting possible base-assisted non-radiative relaxation pathways that may involve possible PET interactions. In contrast, an increase in emission intensity was observed in viscous media (DMSO–glycerol mixture), consistent with viscosity-enhanced fluorescence arising from restriction of intramolecular motion (RIM). Collectively, these findings demonstrate that the introduction of a fluorene spacer into triazine-BODIPY frameworks enables environment-responsive fluorescence, providing new design insights for environment-responsive fluorophores with improved structural rigidity and stable emission behavior.

## Abstract 2

A star-shaped triazine-BODIPY trimer incorporating fluorene spacers was successfully synthesized and well characterized. The compound exhibits aggregation-induced quenching in water, and increased emission intensity in viscous media.

## Graphical abstract

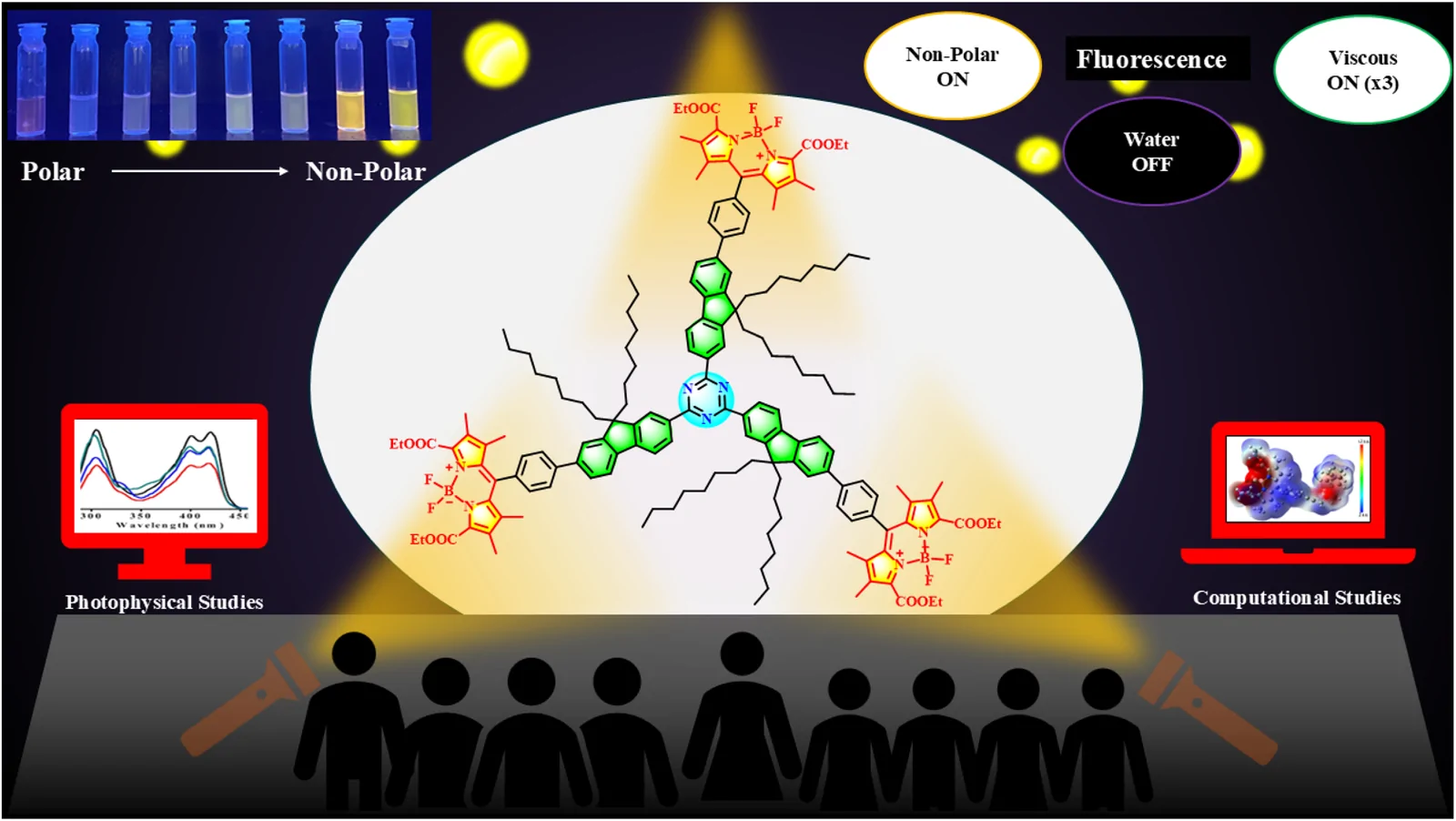

## 1. Introduction

Organic fluorophores with tunable excited-state properties underpin progress in biological imaging, chemical sensing, photodynamic therapy, and optoelectronic devices.^1–3^ Among these, boron-dipyrromethene (BODIPY) dyes have emerged as a privileged class owing to their high molar absorptivity, sharp emission bands, excellent photostability, and synthetic versatility.^4–6^ BODIPY-based compounds have garnered significant interest due to their versatility across various applications, including drug administration,^7–10^ bioimaging,^11,12^ photodynamic treatment^13,14^ (PDT), and artificial photosynthetic systems.^15–17^ Structural engineering of BODIPY frameworks has enabled fine-tuning of their optical and physicochemical properties.

Fluorene is a polycyclic aromatic hydrocarbon characterized by two fused benzene rings attached to a central five-membered ring (Fig. 1). It features a rigid, nearly planar structure, along with high fluorescence quantum yield, strong photostability, and effective hole-transporting properties.^18–21^ These properties have made it a widely used π-bridge in functional optoelectronic materials,^22–24^ and several fluorene-BODIPY conjugates have been reported for near-infrared emission, solar energy conversion and panchromatic absorption.^25–27^ Complementarily, 1,3,5-triazine (*s*-triazine) is a symmetrical, electron-deficient heterocycle whose rigid C_3_-symmetric architecture and thermal robustness make it a convenient core for star-shaped molecular assemblies.^28–31^ It can be readily synthesized by triflic acid-mediated cyclotrimerization of aromatic nitriles.^32^ The combination of these motifs with BODIPY is of particular relevance to multichromophoric design. Triazine-bridged BODIPY dyads and tripods support efficient energy transfer and interchromophore communication,^27,28^ while star-shaped triazine-cored systems bearing fluorene arms display aggregation-induced emission and enhanced two-photon absorption relative to their monomeric analogues.^29,33^ Such architectures are appealing because the close spatial arrangement of multiple chromophores around a central core leads to collective effects like cooperative self-assembly, altered exciton behavior, and enhanced optical responses, which cannot be achieved with a single BODIPY unit.

**Fig. 1:**
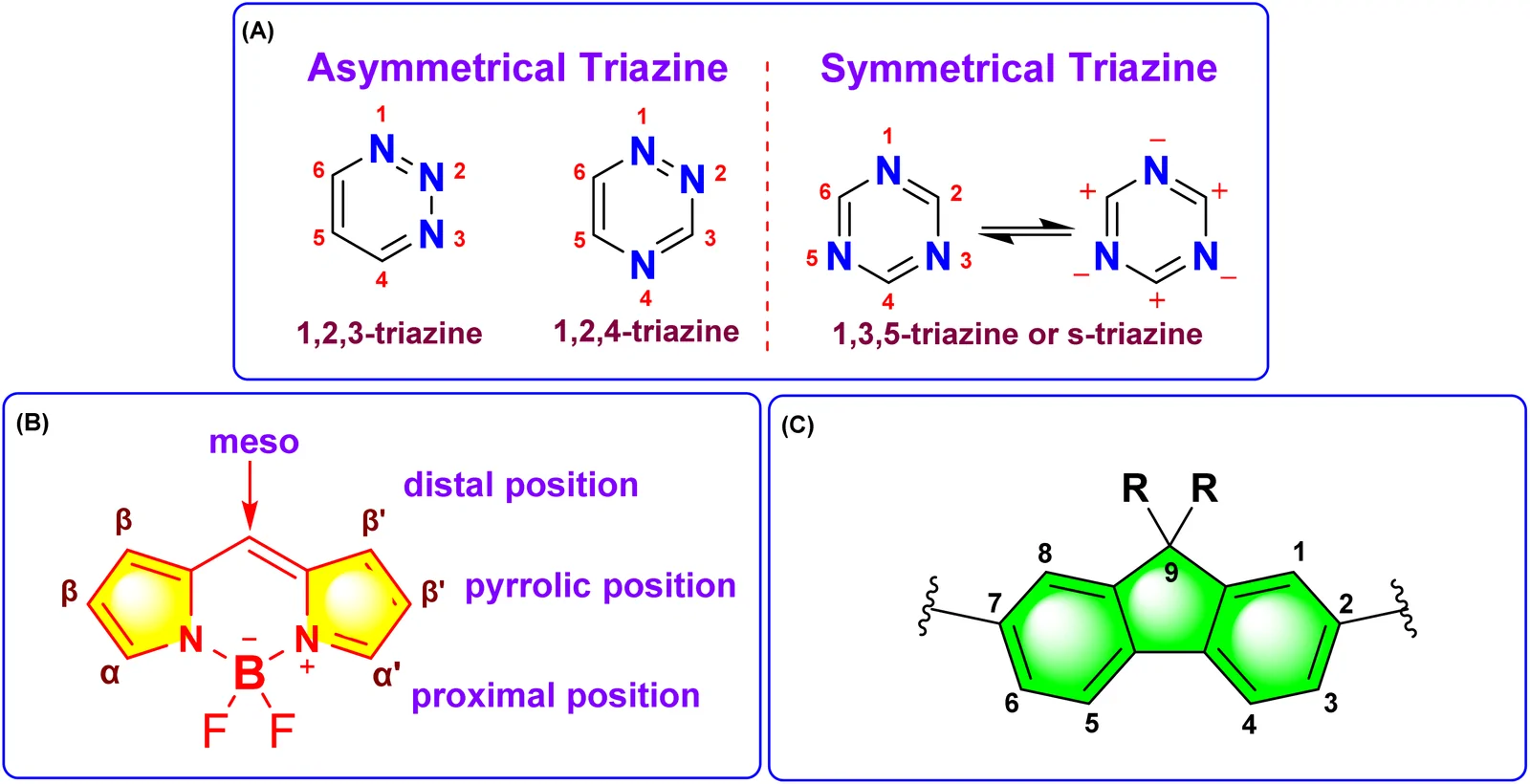
(A) Different types of triazines; (B) different substitution positions of BODIPY; (C) structure of the fluorene spacer used in the present study.

A central challenge in fluorophore design lies in understanding how extrinsic factors such as solvent polarity, hydrogen bonding, viscosity, and aggregation determine the balance between radiative and nonradiative deactivation pathways.^31,34,35^ Integrating *s*-triazine and BODIPY frameworks provides a flexible foundation for developing conjugated systems with adjustable excited-state characteristics. The spacers significantly influence electronic communication between donor and acceptor units, as well as the chromophore's behavior in solution. Among commonly employed π-spacers, simple phenylene bridges permit free rotation, resulting in torsional disorder that reduces π-orbital overlap and opens low-frequency nonradiative decay channels; thiophene-based linkers, while effective at narrowing the donor–acceptor gap, introduce a heavy-atom-like sulfur environment that can enhance intersystem crossing at the expense of fluorescence quantum yield and photostability,^36^ both undesirable for an emissive, environment-responsive probe. In this work, the 9,9-dioctylfluorene unit was chosen as the spacer because its fused biphenyl core is naturally locked into a coplanar and rigid structure, which extends π-conjugation without needing rotation. The 9,9-dialkyl substitution further improves solubility without affecting this planarity.^37,38^ This configuration facilitates electronic communication between the donor and acceptor while constraining low-frequency torsional motion around the spacer, thereby reducing aggregation-induced non-radiative decay. The trimeric structure was chosen over a single BODIPY to investigate how three chromophores, arranged in a fixed relative orientation around an electron-deficient core, behave together in various microenvironments that a monomeric analog cannot demonstrate.

Herein, we report the synthesis and comprehensive photophysical investigation of a star-shaped triazine-BODIPY trimer incorporating fluorene spacers (Tz-Fl-BDP). The study involved detailed structural and electronic characterization, supported by density functional theory (DFT) calculations to understand the electronic distribution and charge-transfer behavior of the molecule. The photophysical properties were systematically examined through absorption and fluorescence spectroscopy across solvents of varying polarity. Steady-state and time-resolved fluorescence measurements were performed to probe the excited-state dynamics, while dynamic light scattering (DLS) analyses were conducted to evaluate aggregation behavior in different solvent environments. In addition, the fluorescence response of Tz-Fl-BDP was studied under varying microenvironmental conditions, including changes in acidity, basicity, and viscosity, to explore the influence of external stimuli on its emission characteristics. Together, these experimental and computational studies provide a comprehensive understanding of the molecular structure, photophysical properties, and environment-dependent behavior of Tz-Fl-BDP.

## 2. Result and discussion

### 2.1 Synthesis

The synthetic pathway for the synthesis of triazine-BODIPY trimer with fluorene spacer (Tz-Fl-BDP) is outlined in Scheme 1.

**Scheme 1:**
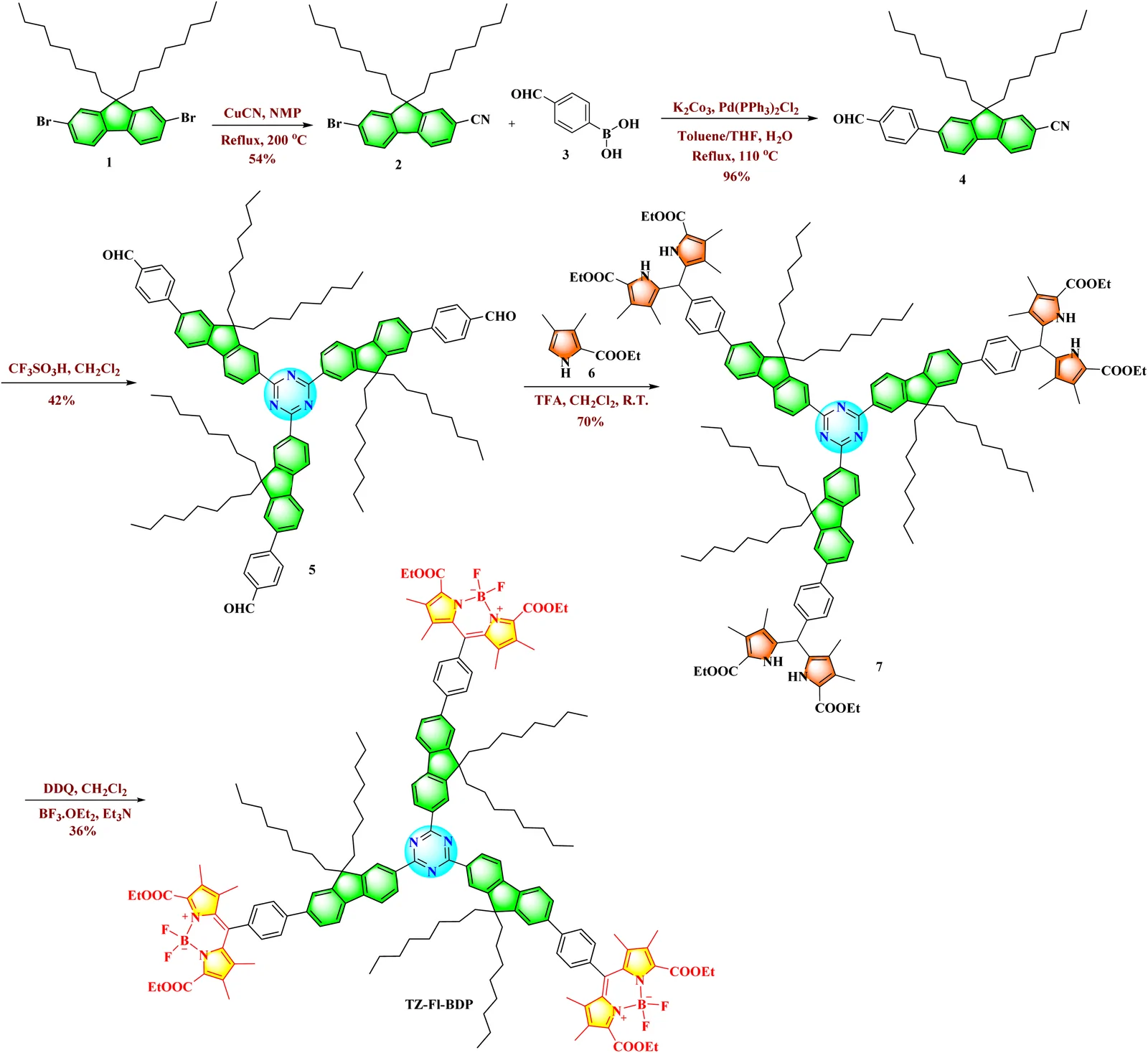
Synthetic route for Tz-Fl-BDP.

The synthesis of Tz-Fl-BDP was initiated by mono-cyanation of 2,7-dibromo-9,9-dioctylfluorene 1 with cuprous cyanide under Rosenmund-von Braun conditions to give compound 2.^33^ Compound 2 undergoes the Suzuki–Miyaura coupling reaction^39^ with 4-formylphenylboronic acid 3 in the presence of a palladium catalyst and K_2_CO_3_, producing compound 4 as a colorless solid with a 96% yield. Compound 4 undergoes a trimerization reaction^39,40^ in the presence of trifluoromethanesulfonic acid to obtain 5 as a white powder. Compound 5 undergoes acid-mediated condensation^41–43^ with 3,4-dimethyl-2-carboxypyrrole 6 in the presence of a catalytic amount of TFA, producing symmetrical triazine-cored dipyrromethane 7 as a colorless solid with a 70% yield. The above-mentioned substituted pyrrole 6 was synthesized according to the literature procedures in our laboratory.^44^ Compound 7 undergoes oxidation with DDQ, followed by the subsequent addition of triethylamine and immediately followed by the addition of BF_3_·OEt_2_, forming the desired Tz-Fl-BDP as a red solid in 36% yield (Scheme 1). Compound Tz-Fl-BDP was characterized by mass spectrometry, NMR, and IR spectroscopy. The IR spectra exhibited a characteristic B–F stretching frequency at 1177 cm^−1^ and a B–N stretching frequency at 1378 cm^−1^. The ^11^B and ^19^F NMR spectra confirmed the presence of a BF_2_ unit in BODIPY. The proton-decoupled ^11^B NMR spectra indicate the presence of boron, with a triplet signal found at *δ* −0.60 ppm. The ^19^F NMR spectra verified the presence of fluorine with a quartet signal at *δ* −141.18. In ^1^H NMR spectra, the removal of the NH proton signal at *δ* 8.56 ppm and the meso proton signal at *δ* 5.57 ppm of compound 7 confirms the synthesis of compound Tz-Fl-BDP. Finally, the MALDI-TOF mass spectral analysis showed a peak at *m*/*z* 2644.6131 [M^+^] for compound Tz-Fl-BDP, confirming its formation.

### 2.2 Density functional theory (DFT) calculations

To thoroughly understand the electronic properties of Tz-Fl-BDP, density functional theory (DFT) calculations were performed using the B3LYP hybrid functional with the 6-311++G(d,p) basis set, incorporating solvent effects *via* the PCM model for dichloromethane. The optimized molecular structure is shown in Fig. S17.

#### 2.2.1 Frontier molecular orbital analysis

Frontier molecular orbital (FMO) analysis of Tz-Fl-BDP was carried out to examine the spatial distribution and energetics of the frontier orbitals (Fig. 2). The three peripheral BODIPY units are electronically near-equivalent: the HOMO, HOMO−1 and HOMO−2 span only 0.04 eV, as do the LUMO, LUMO+1 and LUMO+2 (Table 1), reflecting three symmetry-equivalent arms that behave as essentially independent chromophores. The frontier orbitals are therefore shown localised on a single representative arm. The HOMO and LUMO energy for Tz-Fl-BDP were determined to be −6.09 eV and −3.31 eV, respectively (Table 1). Consequently, the Δ*E*_g_ values for Tz-Fl-BDP are calculated to be 2.79 eV. Interestingly, both the HOMO and LUMO orbitals are primarily localized on the BODIPY core, which is consistent with the literature.^45,46^

**Fig. 2:**
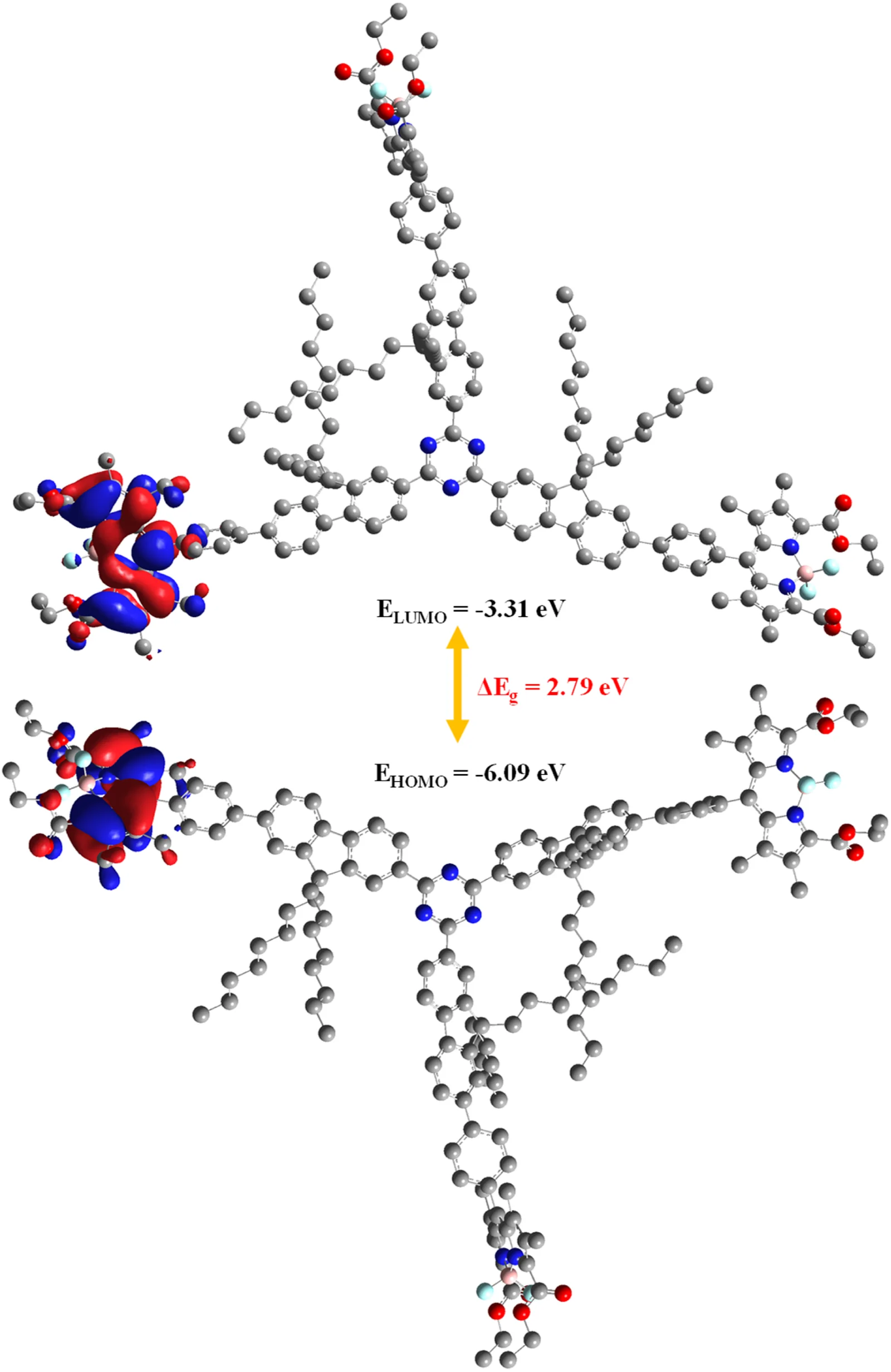
Theoretically calculated HOMO–LUMO energy gap and surface map of Tz-Fl-BDP.

**Table 1:** Computed energies of the frontier molecular orbitals of Tz-Fl-BDP at B3LYP/6-311++G(d,p) level of theory (unit is in eV)

Substrate	Computed energies (in eV) of the frontier molecular orbitals							
	H	H−1	H−2	H−3	L	L+1	L+2	L+3
Tz-Fl-BDP	−6.09	−6.11	−6.13	−6.22	−3.31	−3.27	−3.27	−2.41

#### 2.2.2 TD-DFT excited-state analysis

Time-dependent density functional theory (TD-DFT) calculations were carried out using the B3LYP functional with the 6-311++G(d,p) basis set, incorporating solvent effects through the polarizable continuum model (PCM) with dichloromethane (DCM) as the solvent, to investigate the electronic excitation behaviour of Tz-Fl-BDP. A total of 30 singlet excited states were computed. The simulated absorption spectra are presented in Fig. S18, and the principal excitation parameters are summarized in Table 2.

**Table 2:** TD-DFT-calculated electronic absorption properties of Tz-Fl-BDP at the B3LYP/6-311G ++ G(d,p) level of theory using PCM (DCM) as solvent

Molecules	S_*n*_	Energy (eV)	*λ* (nm)	*f*	Dominant transition
Tz-Fl-BDP	S_1_	2.4047	515.59	0.0444	H → L
	S_2_	2.4119	514.05	0.0025	H−1 → L+1
	S_3_	2.4336	509.46	0.0007	H → L+2
	S_4_	2.6509	467.71	0.3722	H−3 → L
	S_5_	2.6516	467.59	0.3538	H−3 → L
	S_6_	2.6543	467.11	0.0004	H−1 → L+1
	S_7_	2.6552	466.95	0.0000	H−1 → L
	S_8_	2.6562	466.77	0.4510	H−4 → L+2
	S_9_	2.6612	465.90	0.0001	H → L+2
	S_10_	2.6792	462.77	0.0000	H → L+2

The calculated spectrum reveals multiple electronic transitions spanning the visible to near-UV region (≈515–365 nm). The lowest-energy transition (S_1_) appears at 515.6 nm (2.4047 eV) with a modest oscillator strength (*f* = 0.044). Orbital analysis reveals that this excitation arises predominantly from HOMO → LUMO (49%) and HOMO−1 → LUMO (47%) excitations. Importantly, both the HOMO and HOMO−1 are localized on the BF_2_-BODIPY peripheral units, and the LUMO likewise resides on the BODIPY acceptor arms; S_1_ therefore corresponds to a local π → π* excitation within the BODIPY chromophores rather than an ICT transition. Appreciable donor–acceptor charge-transfer character is carried by the prominent absorption cluster near 467 nm comprising S_4_ (*f* = 0.372), S_5_ (*f* = 0.354), and S_8_ (*f* = 0.451). These transitions are dominated by excitations from HOMO−3 and HOMO−4, which are delocalized over the electron-rich triazine–fluorene central donor scaffold, into the BODIPY-centred LUMO and LUMO+1 (Fig. S19). The spatial separation between the hole (donor core) and the electron (BODIPY acceptor arms) in these transitions constitutes direct computational evidence for a D–π–A ICT process, as further supported by the HOMO−3 and LUMO iso-surface maps presented in Fig. 3. At higher energies, moderately intense bands occur near 408 nm (S_19_–S_21_, *f* ≈ 0.24–0.28), followed by a very strong absorption at 386.7 nm (S_22_) with an exceptionally large oscillator strength (*f* = 1.41), which involves transitions from deep occupied orbitals into the higher LUMO manifold and constitutes the higher-energy intense absorption band of the molecule. Several intervening excited states exhibit negligible oscillator strengths (*f* ≈ 0), indicating symmetry-forbidden or weakly allowed transitions unlikely to contribute significantly to the experimental absorption spectrum.

**Fig. 3:**
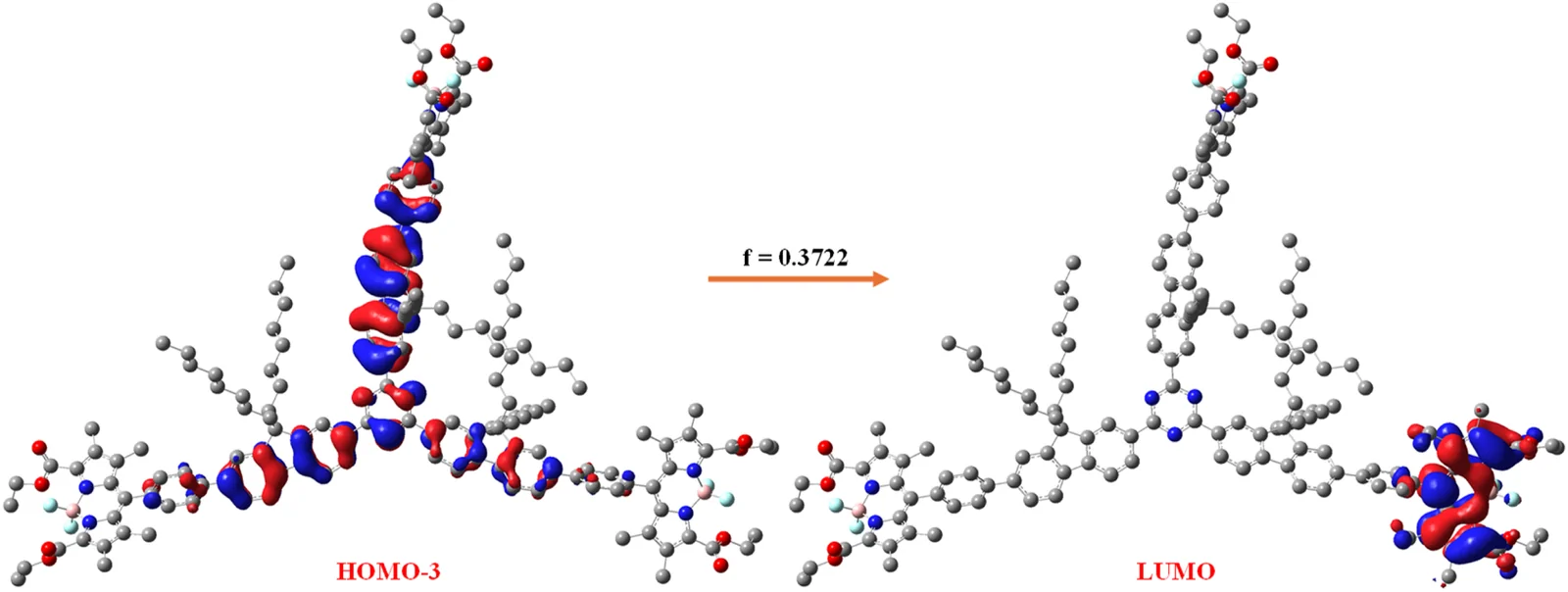
HOMO−3 and LUMO iso-surface maps of Tz-Fl-BDP responsible for ICT transition.

Comparison with the experimental absorbance shows a consistent and interpretable pattern. The computed S_1_ state (515.6 nm, 2.4047 eV) reproduces the experimental low-energy band at 530 nm, in line with the generally reliable performance of B3LYP for locally excited π–π* transitions. By contrast, the computed CT-active cluster (S_4_/S_5_/S_8_, 466.8–467.7 nm) is displaced from the experimental higher-energy band at ∼417 nm by roughly 50 nm, with the computed excitation energies being systematically underestimated. This is the behavior expected of a conventional hybrid functional for charge-transfer states, arising from residual self-interaction error and insufficient long-range Hartree–Fock exchange, and a range-separated functional such as CAM-B3LYP or ωB97X-D would be expected to reduce the discrepancy.

### 2.3 Photophysical studies

#### 2.3.1 UV-visible studies in the solution phase

The UV-visible absorption spectra of the Tz-Fl-BDP were recorded in a broad set of organic solvents spanning a wide polarity range, moderately polar solvents such as toluene (*E*_T_^30^ ≈ 34–38 kcal mol^−1^, *α* = 0.0, *β* = 0.1–0.4), to highly polar aprotic media including acetonitrile and DMSO (*E*_T_^30^ ≈ 45–46 kcal mol^−1^, *α* ≈ 0.0–0.2, *β* ≈ 0.4–0.8), and finally to protic solvents such as methanol (*E*_T_^30^ ≈ 55 kcal mol^−1^, *α* ≈ 0.93, *β* ≈ 0.62) and water (*E*_T_^30^ ≈ 63 kcal mol^−1^, *α* ≈ 1.17, *β* ≈ 0.47). Irrespective of the solvent environment, two major absorption bands were consistently observed near 417 and 530 nm. TD-DFT calculations indicate that the lower-energy ∼530 nm band originates predominantly from localized BODIPY-centered π → π* transitions corresponding to the S_1_ manifold, whereas the higher-energy ∼417 nm band contains significant ICT character arising from electron redistribution between the triazine–fluorene donor scaffold and the peripheral BODIPY acceptor units. The weak solvent dependence of the ∼530 nm band (∼7 nm shift) further supports its primarily localized excited-state nature (Fig. 4). However, distinct deviations were observed in two cases: in toluene, a sharp red-shifted maximum at ∼536 nm indicated the possible formation of a weak charge-transfer complex between the π-rich aromatic ring of toluene and the electron-deficient triazine unit of the TZ-Fl-BDP; in water, a similarly red-shifted band at ∼537 nm appeared but with broadening and residual long-wavelength absorption, consistent with aggregation and self-assembly induced by hydrophobic interactions and π–π stacking of the Tz-Fl-BDP. The broad spectral profile and extended tailing observed in water are consistent with aggregation-associated changes in the photophysical behaviour of Tz-Fl-BDP.^47,48^

**Fig. 4:**
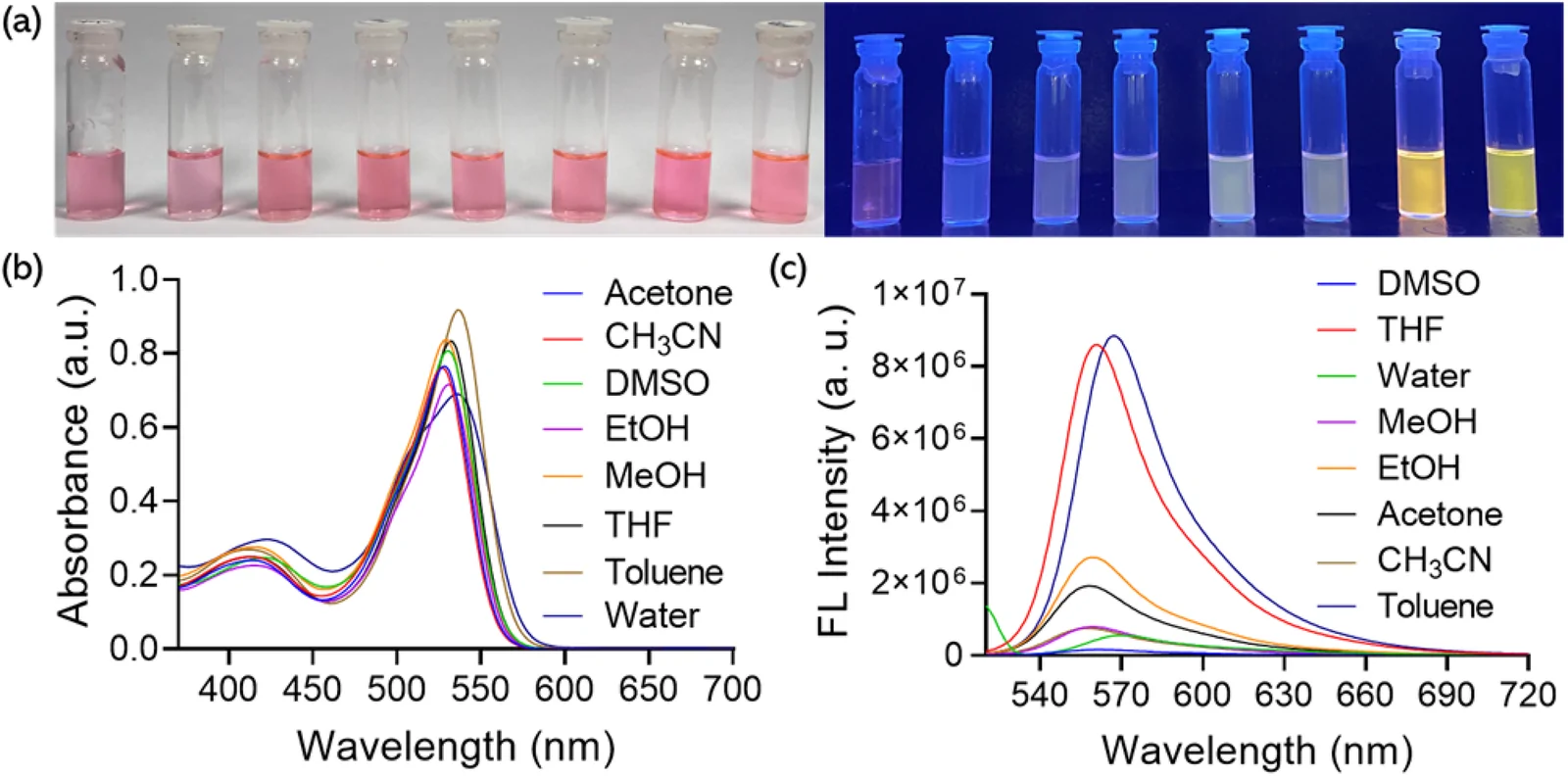
(a) Images of the TZ-Fl-BDP in different organic solvents and water medium under normal daylight (left) and UV light (right), water, DMSO, CH_3_CN, MeOH, ethanol, acetone, toluene, and THF (left to right). (b) UV-visible spectra of the TZ-Fl-BDP (10 µM) in different organic solvents and water medium. (c) Fluorescence spectra of TZ-Fl-BDP (10 µM, *λ*_ex_ = 500 nm) in different organic solvents and in a water medium.

#### 2.3.2 Steady-state fluorescence studies in the solution phase

The fluorescence spectra exhibited complementary solvent-dependent behavior. Strong emission was observed in nonpolar to moderately polar solvents, with maxima at 560 nm in THF and 567 nm in toluene, where weak solute–solvent interactions favor radiative decay. The particularly high intensity and red-shifted maximum in toluene are consistent with the absence of hydrogen-bonding and dipolar relaxation pathways in a nonpolar aromatic medium, which leaves the locally excited state largely unperturbed and favours radiative decay.^49^ In acetone and ethanol, moderate emission was recorded around 560 nm with slight broadening, reflecting an increased contribution from non-radiative deactivation associated with hydrogen bonding and solvent-dependent aggregation. In contrast, polar solvents such as acetonitrile and methanol (*λ*_em_ ≈ 557 nm) produced markedly weaker fluorescence. Notably, the emission maximum remains essentially invariant (≈557–572 nm) across the full polarity range examined, so this quenching is attributed to solvent-promoted non-radiative decay, principally hydrogen bonding and dielectric relaxation acting on a locally excited state, rather than to progressive stabilization of a charge-transfer state. In DMSO, the pronounced quenching observed arises primarily from its high polarity and strong solvation ability, which promote dielectric stabilization of the excited state and enhance non-radiative relaxation, rather than from direct hydrogen-bonding interactions. In water, the emission maximum was further red-shifted to 572 nm with intensity comparable to methanol, a behaviour consistent with aggregation-induced quenching in self-assembled aggregates, which accounts for both the red-shift and the broadening.^50,51^ Collectively, these results demonstrate that while the star-shaped triazine-BODIPY trimer with fluorene spacer (Tz-Fl-BDP) shows only modest solvatochromism in absorption, its fluorescence is highly sensitive to the combined effects of solvent polarity, hydrogen-bonding interactions, and aggregation in aqueous media.

The fluorescence excitation spectra recorded in THF closely matched the absorption profile, while the emission maximum remained nearly invariant across the solvents examined. Together with the relatively small Stokes shifts (975–1369 cm^−1^, Table 3), these observations are consistent with emission from a locally excited (LE) state localized on the dipyrromethene π-system of the BODIPY chromophore rather than from a relaxed ICT state. The observed emission at ∼560 nm is red-shifted relative to typical BODIPY emission (510–530 nm)^52^ and can be associated with the electronic influence of the CO_2_Et-substituted BODIPY framework. The S_1_ state of BODIPY is a π → π* transition confined largely to the dipyrromethene π-system, so its energy is governed by the HOMO–LUMO separation, and the 3- and 5-positions lie at the termini of this π-system. Substituents at these positions are therefore strongly conjugatively coupled to the chromophore. The ethoxycarbonyl group is a π-acceptor, and mixing of its low-lying π*(C=O) orbital with the BODIPY π* selectively stabilises the LUMO, whereas the HOMO is lowered to a smaller extent through the inductive effect alone. The frontier gap consequently contracts and both absorption and emission shift bathochromically, in agreement with the computed LUMO energy of −3.31 eV. Consistent with this interpretation, the BODIPY architectures we have reported previously that carry the same α-ester substitution all emit near 560 nm irrespective of the core or spacer, indicating that the emission energy is set by the substitution pattern local to the BODIPY unit rather than by the multichromophoric architecture.^45,53,54^ The excitation spectrum of Tz-Fl-BDP in water was broader and deviated from the absorption features, indicating that the emission in aqueous medium originates from aggregated species rather than from molecularly dispersed molecules.^55^ Fluorescence spectra of the Tz-Fl-BDP in water were recorded at different excitation wavelengths (420–520 nm), and in all cases, nearly identical emission profiles were observed, centered at ∼572 nm with no significant spectral shift. This excitation-independent behavior indicates that, regardless of the excitation band, the system relaxes to the same lowest excited singlet state (S_1_) before emission.^56^ The absence of multiple emission bands rules out contributions from both monomeric and aggregated species, suggesting that in aqueous medium monomer emission is negligible and the aggregates act as the sole emissive species.

**Table 3:** Photophysical properties of Tz-Fl-BDP (10 µM) in different solvents, including absorption maximum, molar extinction coefficient, emission maximum, Stokes shift, fluorescence lifetime, fluorescence quantum yield, radiative, and non-radiative rate constants

Solvent	*λ*_abs_ (nm) [*ε*] (L mol^−1^ cm^−1^)	*λ*_em_ (nm)	Stokes shift (cm^−1^)	Lifetime (ns)	*Φ*_f_ (%)	*k*_r_ (10^8^ s^−1^)	*k*_nr_ (10^8^ s^−1^)
Water	535 [68 100]	570	1147	0.367	4.4	1.21	26
DMSO	530 [80 000]	566	1200	0.441	1.2	0.28	22
ACN	527 [76 000]	560	1118	0.588	4.7	0.81	16
MeOH	529 [83 600]	566	1235	0.614	4.4	0.72	15
EtOH	531 [71 200]	560	975	0.761	18	2.39	10
Acetone	527 [75 400]	568	1369	0.631	12	1.99	13
Toluene	535 [91 300]	567	1054	1.925	53	2.80	2.3
THF	530 [82 500]	560	1010	2.25	55	2.46	1.9

The fluorescence behavior of the Tz-Fl-BDP in THF was systematically monitored upon gradual addition of water and methanol to probe the effect of increasing solvent polarity and hydrogen-bonding strength (Fig. 5). Upon stepwise addition of water, a pronounced quenching of fluorescence intensity was observed, with ∼20-fold reduction at high water fractions, accompanied by a clear red shift of the emission maximum from 560 to 572 nm. The red-shifted and broadened profile, together with the large decrease in intensity, strongly suggests aggregation-induced quenching (AIQ) triggered by the poor solubility of the Tz-Fl-BDP in aqueous medium.^57^ Hydrophobic and π–π stacking interactions drive the formation of supramolecular assemblies; AIQ associated with the formation of self-assembled molecular aggregates between closely packed Tz-Fl-BDP alters the excited-state dynamics, producing red-shifted, broadened, and weakened emission. The residual emission is therefore attributed to aggregated species rather than isolated molecular fluorescence. In contrast, titration with methanol resulted in a ∼10-fold quenching of fluorescence without any noticeable spectral shift, with the emission maximum remaining at ∼560 nm. This indicates that in THF–MeOH mixtures, the Tz-Fl-BDP remains largely monomeric, but enhanced solute–solvent hydrogen bonding and dielectric stabilisation of the excited state increase non-radiative decay pathways, thereby reducing fluorescence intensity. Similarly, the absorption spectra recorded during THF–water titration show an increase in tailing and absorbance near 560 nm with increasing THF fraction, consistent with aggregation-induced spectral broadening. In contrast, very little change was observed in THF–MeOH mixtures, confirming that aggregation effects are minimal in methanol-rich media. The corresponding *A*/*A*_0_*vs.* % THF plot is shown in Fig. S19. The absence of a spectral shift in methanol mixtures confirms that aggregation does not play a significant role, and the quenching arises primarily from dynamic solute–solvent interactions. DLS studies revealed aggregates in all solvents, with the largest hydrodynamic diameters observed in water. The strong hydrophobic effect and the poor solubility of Tz-Fl-BDP in aqueous medium promote extensive self-association, which correlates with the enhanced aggregation-assisted quenching observed. In contrast, the aggregates detected in THF appear optically inactive, producing no significant spectral perturbations.

**Fig. 5:**
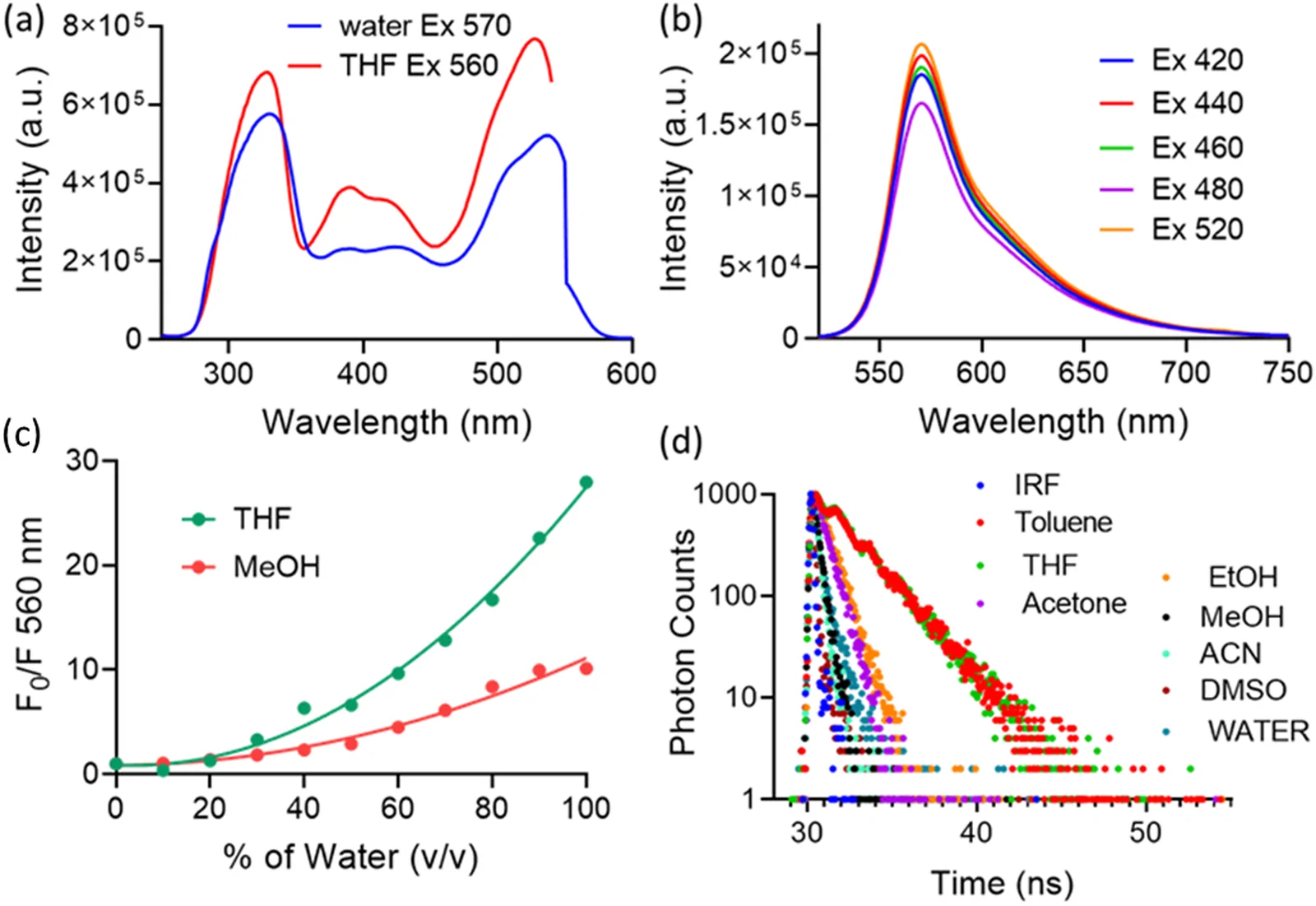
(a) Fluorescence excitation spectra of Tz-Fl-BDP (10 µM, *λ*_ex_ = 500 nm) in THF and water medium (at respective emission maxima). (b) Fluorescence spectra of Tz-Fl-BDP (10 µM) in water medium at different excitation channels. (c) Changes in fluorescence intensity of Tz-Fl-BDP (10 µM, *λ*_ex_ = 500 nm) at 560 nm in different THF–H_2_O and THF–MeOH mixtures. (d) Fluorescence decay spectra of Tz-Fl-BDP (10 µM, *λ*_ex_ = 500 nm) in different organic solvents and water medium (at respective emission maxima).

#### 2.3.3 Time-resolved fluorescence studies in the solution phase

Time-resolved fluorescence measurements provided further insight into the excited-state dynamics and complemented the steady-state observations (Table 3). In nonpolar and weakly polar solvents such as THF and toluene, the Tz-Fl-BDP exhibited the longest average lifetimes (*τ*_avg_ ≈ 1.8–2.2 ns), which correlate with their strong emission intensities. The extended lifetimes here arise from the dominance of radiative decay pathways and the absence of significant solute–solvent interactions that could open up non-radiative channels.^58^ The weak donor–acceptor interaction can also rationalize the particularly long lifetime in toluene between the π-rich solvent and the triazine unit of the molecule: this stabilizes the excited state without significantly increasing non-radiative relaxation, thereby preserving efficient emission from the LE state. In acetone and ethanol, intermediate lifetimes (∼0.6–0.7 ns) were observed, consistent with their moderate fluorescence intensities (Fig. 6). In these cases, increased solvent polarity facilitates solvent-assisted non-radiative relaxation of the excited state, thus increasing the non-radiative decay rate while still allowing a fraction of radiative emission. The effect of hydrogen bonding is also evident in ethanol, where dynamic solute–solvent interactions lead to spectral broadening and a reduction in lifetime relative to THF. On the other hand, in highly polar solvents such as acetonitrile and methanol, the lifetimes shortened dramatically (*τ*_avg_ ≈ 0.5–0.6 ns). This reflects strong solvation of the excited state, which lowers the energy gap between the excited and ground states, thereby enhancing internal conversion and vibrational relaxation. In methanol, the strong hydrogen-bond donor ability (*α* ≈ 0.93) introduces an additional non-radiative deactivation pathway through dynamic hydrogen-bonding with the fluorophore, further accelerating excited-state decay. The most striking quenching was observed in DMSO, where the average lifetime was reduced to ∼0.4 ns, much shorter than in toluene. This can be explained by the combined effects of high polarity and very strong hydrogen-bond-accepting ability (*β* ≈ 0.76) of DMSO, which promotes ultrafast non-radiative relaxation through solvent-induced stabilization of the excited state and solute–solvent vibrational coupling, effectively suppressing fluorescence. Interestingly, in water, the compound exhibited a moderately short but non-negligible lifetime (∼0.3 ns), coupled with red-shifted emission at 570 nm. This behavior deviates from the trend in alcohols and can be critically attributed to aggregation-induced effects: hydrophobic interactions in water drive self-assembly of the Tz-Fl-BDP into π–π stacked aggregates, which introduce aggregate-derived emissive states associated with molecular aggregation.^59^ These aggregate states decay faster than the monomer emission in THF or toluene, but slower than the strongly quenched states in methanol or DMSO, producing an intermediate lifetime with reduced intensity and a characteristic red shift.

**Fig. 6:**
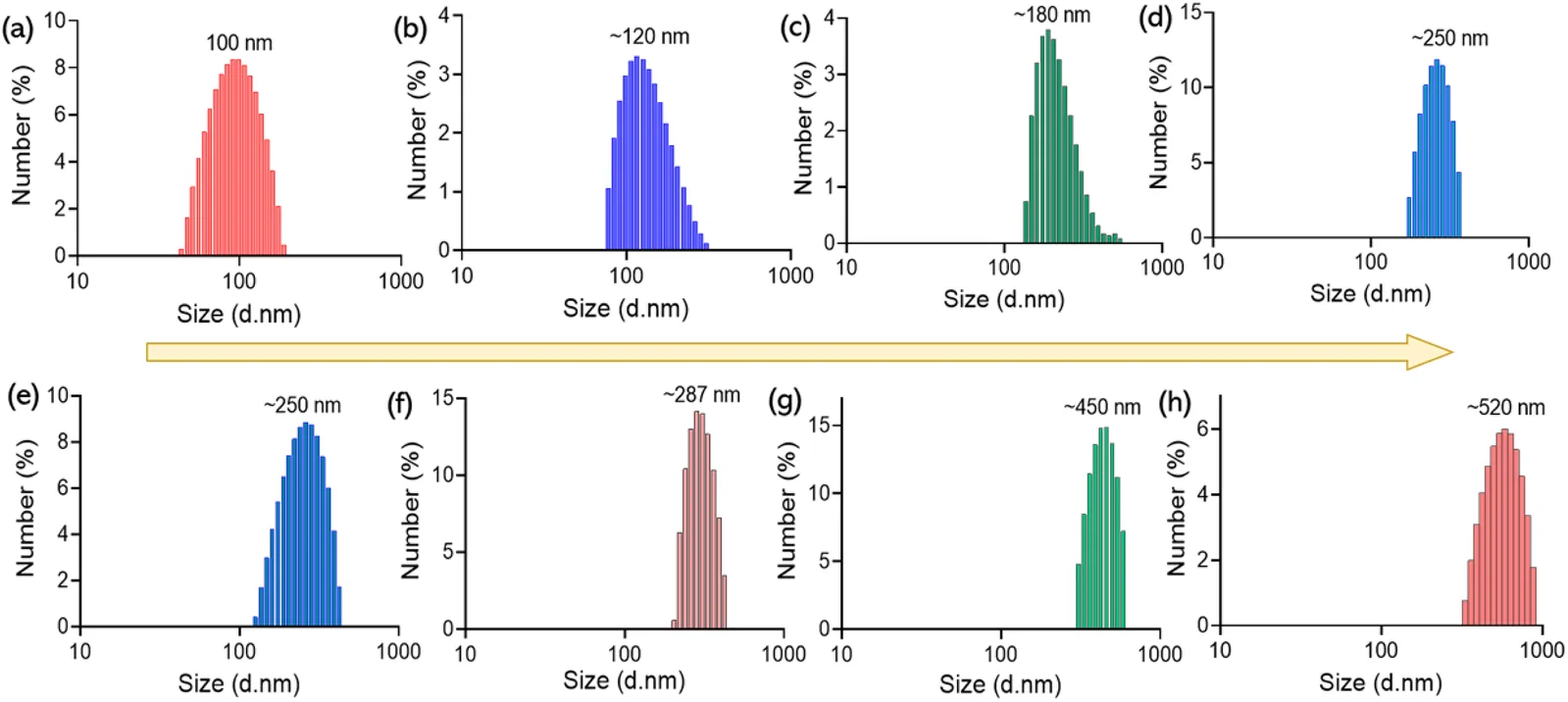
Determination of hydrodynamic diameter of Tz-Fl-BDP in (a) THF, (b) toluene, (c) ethanol, (d) acetone, (e) DMSO, (f) CH_3_CN, (g) methanol, (h) H_2_O.

#### 2.3.4 Self-assembly studies in solution phase

The DLS results strongly support the solvent-dependent photophysical observations. In nonpolar/weakly polar solvents, such as THF or toluene, size distributions were relatively narrow and centered at smaller diameters (100–120 nm), indicating that the compound exists mostly as molecularly dispersed species or small nano-aggregates. The absence of broad secondary peaks confirms good solubility and stable dispersion. In moderately polar solvents such as ethanol, acetone, and acetonitrile (180–290 nm), broader distributions appeared with peaks slightly shifted toward higher diameters. This suggested partial aggregation due to polarity mismatch, where the Tz-Fl-BDP is less soluble and tends to self-associate *via* π–π stacking and hydrophobic interactions. In DMSO, we observed broader distribution skewed toward higher diameters (∼250 nm). Even though DMSO is miscible and highly polar, its strong H-bond acceptor property (*β* ≈ 0.76) might interact with peripheral groups, disrupting solvation of the aromatic core and encouraging aggregation. In the case of water and MeOH, distribution became broad and shifted to much larger hydrodynamic diameters (∼520 and 450 nm, respectively). This indicated pronounced self-assembly and aggregate formation, driven by hydrophobic collapse and hydrogen bonding of solvent with the triazine-cored BODIPY molecules. Aggregates likely adopted π–π stacked or micelle-like supramolecular assemblies. This also explained the red-shifted absorption/emission, broadening, and aggregation-induced quenching seen in photophysical studies.

#### 2.3.5 Effect of microenvironment on fluorescence response in solution phase

The fluorescence response of the Tz-Fl-BDP was further evaluated under acidic and basic conditions by adding trifluoroacetic acid (TFA) and triethylamine (TEA), respectively. Interestingly, no significant change in emission profile or intensity was observed upon addition of TFA, suggesting that protonation of the triazine or peripheral donor groups is either energetically unfavourable or does not significantly perturb the excited-state electronic structure responsible for LE emission of the Tz-Fl-BDP. This insensitivity to acidic conditions may arise from the electron-deficient and rigid triazine-cored BODIPY framework, in which frontier orbital distribution is not strongly modulated by protonation, thereby preserving both absorption and emission characteristics. In contrast, upon addition of TEA, a moderate fluorescence quenching of ∼1.47-fold was observed, without any spectral shift (Fig. 7).

**Fig. 7:**
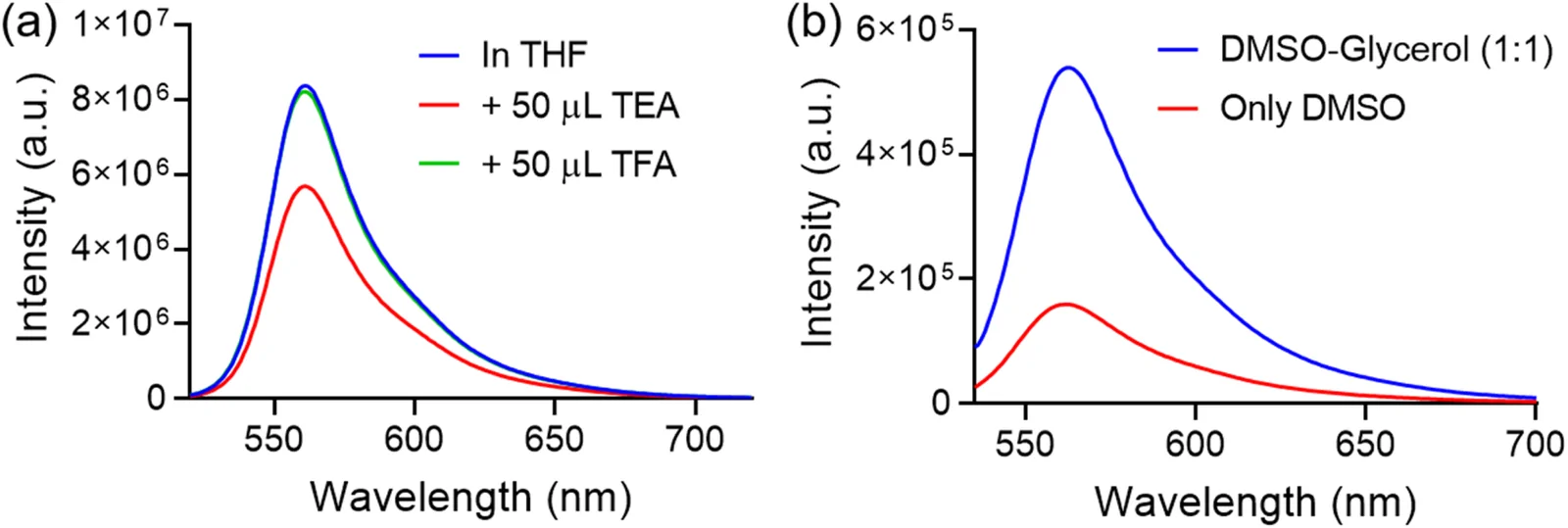
(a) Fluorescence spectra of compound Tz-Fl-BDP (10 µM, *λ*_ex_ = 500 nm) in THF medium upon addition of acid (TFA) and alkali (TEA). (b) Fluorescence spectra of Tz-Fl-BDP (10 µM, *λ*_ex_ = 500 nm) in DMSO and DMSO–glycerol (1 : 1) mixture medium.

The absence of band shift indicates that deprotonation or new emissive species are not formed; instead, quenching likely arises upon TEA addition, suggesting possible base-assisted non-radiative deactivation pathways, which may involve possible PET interactions.^60^ Also, the role of viscosity was examined in DMSO *versus* DMSO–glycerol (1 : 1). In DMSO, fluorescence was almost quenched, while in DMSO–glycerol, intensity increased ∼3-fold with no shift in *λ*_em_. This enhancement can be critically attributed to viscosity-induced restriction of intramolecular motions (RIM) within the flexible triazine-cored BODIPY framework, which suppresses torsional relaxation and other non-radiative decay pathways. Thus, glycerol rescues emission by suppressing molecular motions that are otherwise activated in fluid DMSO.^61^

The radiative (*k*_r_) and nonradiative (*k*_nr_) decay constants were calculated from the experimentally determined fluorescence quantum yields (*Φ*_f_) and average lifetimes (*τ*) using the relations *k*_r_ = *Φ*_f_/*τ* and *k*_nr_ = (1 − *Φ*_f_)/*τ*, which allow separation of the two competing deactivation pathways of the excited state.^62,63^ A clear solvent dependence was observed. In nonpolar solvent like toluene, the Tz-Fl-BDP exhibited the highest radiative rate (*k*_r_ ≈ 2.8 × 10^8^ s^−1^) and longest lifetime (*τ* ≈ 1.92 ns), yielding an appreciable quantum yield (*Φ*_f_ = 53%), which reflects the dominance of radiative decay under conditions where solute–solvent interactions are minimal and weak excited-state stabilization by π–π interactions with the solvent is beneficial. In ethanol, *k*_r_ was lower (2.39 × 10^8^ s^−1^) and *k*_nr_ increased to 10 × 10^8^ s^−1^, leading to reduced *Φ*_f_ (18%) despite a reasonably long lifetime (0.76 ns), suggesting that hydrogen bonding introduces additional non-radiative pathways. Acetonitrile, with higher polarity but weaker H-bonding ability, showed a somewhat shorter lifetime (0.588 ns) and reduced *k*_r_ (0.81 × 10^8^ s^−1^), while *k*_nr_ remained significant (16 × 10^8^ s^−1^), consistent with enhanced non-radiative decay in strongly polar media. The Tz-Fl-BDP solution in water displayed the lowest quantum yield (*Φ*_f_ = 4.4%) despite a lifetime comparable to Tz-Fl-BDP solution in acetonitrile (0.367 ns), due to a strongly suppressed radiative rate (*k*_r_ = 1.21 × 10^8^ s^−1^) and very high *k*_nr_ (26 × 10^8^ s^−1^), reflecting dominant aggregation-induced quenching arising from the formation of molecular aggregates in aqueous medium. Thus, analysis of *k*_r_ and *k*_nr_ highlights that solvent polarity, hydrogen-bonding strength, and aggregation cooperatively modulate excited-state deactivation: radiative rates are maximized in nonpolar media, whereas protic and aqueous solvents promote enhanced non-radiative relaxation through solute–solvent interactions and self-assembly.

## 3. Conclusion

In the present research work, a new star-shaped triazine-BODIPY trimer with a fluorene spacer unit (Tz-Fl-BDP) was successfully synthesized and characterized using detailed spectroscopic analyses, including ^1^H, ^13^C, ^11^B, and ^19^F NMR spectroscopy, FTIR spectroscopy, and mass spectrometry. The photophysical properties of this compound were investigated using the above-mentioned techniques in various solvent environments.

The absorption spectra displayed two dominant bands near 417 and 530 nm, corresponding to higher-energy transitions possessing appreciable donor–acceptor charge-transfer character and lower-energy localized BODIPY-centered π → π* transitions, respectively. The weak solvent dependence of both absorption bands indicates that their energies are only modestly perturbed by the medium. Small bathochromic shifts observed in toluene and ethanol are consistent with reduced dipolar stabilisation of the ground state in these solvents. However, in aqueous solution, a broadened, red-shifted absorption peak indicates aggregate formation arising from molecular self-assembly. Fluorescence analyses indicated strong emission in nonpolar solvents (THF, toluene), while significant quenching was observed in highly polar solvents (MeOH, MeCN, DMSO, water). The less efficient emission in these solvents can be attributed to a higher rate of non-radiative decay due to interactions with the solvent, as well as, in water, aggregation-assisted quenching. DLS analyses indicated nm-scale aggregates in all solvents, with the smallest hydrodynamic diameters in THF and toluene (100–120 nm), intermediate values in the moderately polar solvents, and the largest in methanol (∼450 nm) and water (∼520 nm). The higher hydrophobicity and poor solubility of Tz-Fl-BDP in water promotes molecular aggregation, which is consistent with the observed fluorescence quenching. Fluorescence lifetimes measured in the time-resolved fluorescence spectra were solvent-dependent, with maxima in *τ*_avg_ (∼2.0 ns) in nonpolar solvents, while in polar solvents, shorter lifetimes appeared in the range of ∼0.3–0.6 ns. This was compatible with non-radiative relaxation processes. The excitation spectra overlapped with the absorption spectra, thereby disclosing emission from the common excited state. Addition of triethylamine (final concentration 1.25 mM) produced a modest fluorescence quenching (∼1.5-fold) with no change in band position, consistent with the opening of an additional non-radiative deactivation channel. Conversely, emission enhancement in viscous DMSO-glycerol mixtures highlighted restriction of intramolecular motion (RIM) as a governing relaxation pathway under constrained environments. Overall, the excited-state dynamics of Tz-Fl-BDP are modulated jointly by solvent polarity, dipolar stabilization, hydrogen-bonding environment, and aggregation effects. This cooperative influence defines the balance between radiative and nonradiative pathways that establishes Tz-Fl-BDP as an environment-responsive fluorophore with the ability to modulate its optical response across various microenvironments. Such tunable photophysical characteristics hint at its great potential for application in optoelectronic materials, molecular sensing, and solution-phase photochemical systems.

## 4. Experimental

### 4.1 Materials and methods

The chemicals used in this study were sourced from Sigma-Aldrich/Merck India Pvt. Ltd/Spectrochem India and were utilized without further purification. The experiment was carried out using oven-dried glassware inside a dry argon/nitrogen atmosphere. Solvents were distilled under a nitrogen atmosphere by using an appropriate drying agent. The reagents were purchased and utilized without additional purification. The purification of all the compounds was carried out by using silica gel column chromatography (60–120 mesh, Spectrochem, India). JEOL 400 MHz NMR spectrometer (JNM-ECZ400s) was utilized to record the ^1^H, ^13^C, and ^11^B NMR spectra. Chemical shifts were reported with respect to tetramethylsilane (TMS) (^1^H: *δ* = 0.00 ppm) and CDCl_3_ (^13^C: *δ* = 77.0 ppm) as the reference compound, and the coupling constants are given in Hz. All ^13^C spectra are proton-decoupled. ^11^B NMR spectra are proton-decoupled and were recorded at 128 MHz relative to BF_3_·Et_2_O. The infrared (IR) spectra of all compounds were recorded on a Thermo Scientific Nicolet FT-IR spectrophotometer. Mass spectral analyses of newly synthesized compounds were carried out using MALDI-TOF mass spectrometers. Dynamic Light Scattering Studies (DLS) measurements were done using a Malvern Zetasizer NanoZS particle sizer (Malvern Instruments Inc., MA) instrument. Samples were prepared in water and examined under dust-free conditions. Reported mean hydrodynamic diameters were obtained from Gaussian analysis of the intensity-weighted particle size distribution. UV-Vis spectroscopic measurements were conducted on a Shimadzu model 2100 spectrometer. The slit-width used for the experiment was set to 5 nm.

### 4.2 Computational study

All quantum-chemical calculations were performed using the Gaussian 16 (Revision C.01) software package.^64^ The Becke three-parameter hybrid functional combined with the Lee–Yang–Parr correlation functional (B3LYP) and the 6-311++G(d,p) basis set was employed for all geometry optimizations with PCM (DCM) as solvent.^65,66^ Frontier molecular orbital (FMO) analysis was also carried out using this computational approach. Visualizations were generated using GaussView 6.0.^67^

### 4.3 Preparation and analytical data of compounds

#### 4.3.1 Compound 2

Compound 1 (1000 mg, 1.82 mmol) was dissolved in 10 mL of NMP. Cuprous cyanide (146.96 mg, 1.64 mmol) was added under an argon atmosphere. The reaction mixture was then refluxed at 200 °C for 24 hours. Thin-layer chromatography monitored the completion of the reaction. The temperature was subsequently reduced to 120 °C. 1.2 g of FeCl_3_ was dissolved in 2 mL of HCl and 8 mL of water, followed by stirring the reaction mixture for 30 minutes. The flask was then cooled to room temperature. The reaction mixture was extracted with EtOAc. The organic layer was washed with 10% NaOH, 6 N HCl, and water. It was then separated, dried over Na_2_SO_4_, and concentrated using a rotary evaporator to yield the crude product. The crude product was further purified through silica gel column chromatography with hexane as the eluent, resulting in 511 mg of pure product in the form of an oily liquid. Yield: 54%. ^1^H NMR (400 MHz, CDCl_3_): *δ* 7.73 (d, *J* = 8.0 Hz, 1H), 7.64–7.58 (m, 4H), 7.51 (s, 1H), 1.95 (t, *J* = 7.2 Hz, 4H), 1.21–1.04 (m, 20H), 0.81 (t, *J* = 6.8 Hz, 6H), 0.05 (m, 4H); ^13^C NMR (100 MHz, CDCl_3_): 153.41, 150.94, 144.46, 138.04, 131.34, 130.47, 126.39, 126.34, 123.14, 122.04, 120.25, 119.51, 110.32, 55.71, 39.87, 31.62, 29.69, 29.04, 23.56, 22.48, 13.98; IR (neat): 2916, 2846, 2220, 1602, 1455, 1402, 1061, 814 cm^−1^.

#### 4.3.2 Compound 4

Compound 2 (400 mg, 0.808 mmol) was dissolved in 10 mL of a solvent mixture of toluene and THF (1 : 1). To it, 4-formylphenylboronic acid 3 (242.45 mg, 1.617 mmol) was added and stirred at room temperature. Then, Pd(PPh_3_)_2_Cl_2_ (28 mg, 0.04 mmol) was added to the reaction mixture, followed by the addition of K_2_CO_3_ (446 mg, 3.232 mmol) dissolved in 1 mL of distilled water. Then, the reaction mixture was refluxed at 110 °C for 20 h. After completion of the reaction, distilled water was added to the reaction mixture and extracted three times with dichloromethane. Then, the combined organic layer was dried over anhydrous Na_2_SO_4_ and concentrated in a rotary evaporator. After evaporation of the solvent, the crude product was purified by silica gel column chromatography using 4% ethyl acetate in hexane to obtain 401 mg of compound 4 as a white solid, yield: 96%. Mp: 151 °C; ^1^H NMR (400 MHz, CDCl_3_): *δ* 10.08 (s, 1H, –CHO), 7.99 (d, *J* = 8.0 Hz, 2H), 7.84–7.80 (m, 3H), 7.68–7.62 (m, 5H), 2.02 (t, *J* = 7.2 Hz, 4H), 1.24–1.05 (m, 20H), 0.79 (t, *J* = 6.8 Hz, 6H), 0.63–0.57 (m, 4H); ^13^C NMR (100 MHz, CDCl_3_): *δ* 191.84 (–CHO), 152.29, 151.67, 146.97, 144.90, 140.33, 139.50, 135.27, 131.40, 130.29, 127.78, 126.79, 126.48, 121.75, 121.33, 120.50, 119.72, 110.21, 55.68, 40.03, 31.67, 29.77, 29.08, 23.68, 22.52, 14.02; IR (KBr): 2916, 2854, 2730, 2213, 1695, 1602, 1463, 1200, 1169, 814 cm^−1^; HRMS(*m*/*z*): calculated mass for C_37_H_45_NO: 519.3501, found: 537.3942 [M + H_2_O].

#### 4.3.3 Compound 5

Compound 4 (350 mg, 0.673 mmol) was dissolved in 5 mL of dry chloroform. Then, the RB flask was cooled to 0 °C, and CF_3_SO_3_H (0.3 mL, 3.365 mmol) was added dropwise to the reaction mixture. It was stirred at this temperature for 10 minutes and then at room temperature for 24 h. The reaction mixture was quenched with water at 0 °C with a small amount of NH_4_OH. Then, the compound was extracted with dichloromethane. The organic layer was dried over anhydrous Na_2_SO_4_ and concentrated to obtain 441 mg of compound 5 as a white solid. This was used in the next reaction without purification, yield: 42%. Mp: 142 °C; ^1^H NMR (400 MHz, CDCl_3_): *δ* 10.08 (s, 3H, –CHO), 7.98 (d, *J* = 8.0 Hz, 6H), 7.89–7.78 (m, 16H), 7.66–7.61 (m, 6H), 2.07–2.03 (m, 12H), 1.18–1.03 (m, 60H), 0.78 (t, *J* = 6.8 Hz, 18H), 0.63–0.60 (m, 12H); ^13^C NMR (100 MHz, CDCl_3_): *δ* 191.91(–CHO), 169.56, 152.52, 151.58, 147.33, 144.27, 140.31, 139.57, 135.17, 131.94, 130.29, 127.76, 126.56, 126.25, 122.33, 121.75, 120.97, 119.83, 55.58, 40.21, 31.70, 29.88, 29.15, 23.74, 22.54, 14.02; IR(KBr): 3349, 3194, 2916, 2854, 1687, 1656, 1587, 1370, 1208, 1161, 821 cm^−1^. HRMS(*m*/*z*): calculated mass for C_111_H_135_N_3_O_3_: 1558.0503, found: 1558.2425 [M^+^].

#### 4.3.4 Compound 7

Compound 5 (100 mg, 0.064 mmol) and substituted pyrrole 6 (62 mg, 0.384 mmol) were dissolved in dry dichloromethane (5 mL) and stirred at room temperature for 10 min. Then TFA (0.06 mL, 0.64 mmol) was added at 0 °C and stirred at room temperature for 3 h. After the reaction, water was added to the reaction mixture and extracted thrice with dichloromethane. The combined organic layer was dried over anhydrous Na_2_SO_4_ and concentrated in a rotary evaporator. After evaporation of the solvent, the crude product was purified by silica gel column chromatography using 25% ethyl acetate in hexane to obtain 112 mg of compound 7 as a colourless solid. Yield: 70%. Mp: 155 °C. ^1^H NMR (400 MHz, CDCl_3_): *δ* 8.56 (s, 6H), 7.87–7.72 (m, 12H), 7.61–7.52 (m, 12H), 7.18 (d, *J* = 8.0 Hz, 6H), 5.57 (s, 3H), 4.25(q, *J* = 6.8 Hz, 12H, –CH_2ester_), 2.26 (s, 18H, –CH_3_pyrrole), 2.02 (t, *J* = 7.2 Hz, 12H), 1.84 (s, 18H, –CH_3_pyrrole), 1.31(t, *J* = 6.8 Hz, 18H, –CH_3ester_), 1.25–1.03 (m, 60H), 0.78 (t, *J* = 6.8 Hz, 18H), 0.62 (m, 12H). ^13^C NMR (100 MHz, CDCl_3_): *δ* 169.66, 161.90, 152.32, 151.47, 144.65, 140.44, 139.22, 138.21, 131.63, 131.58, 128.69, 127.82, 127.60, 126.23, 126.16, 122.23, 121.42, 120.75, 119.56, 118.06, 117.91, 59.85, 55.47, 40.60, 40.26, 31.71, 29.95, 29.19, 23.81, 22.54, 14.50, 14.01, 10.65, 8.84. IR(KBr): 3295, 2916, 2846, 1656, 1602, 1432, 1255, 1084, 922 cm^−1^. HRMS(*m*/*z*): calculated mass for C_165_H_207_N_9_O_12_: 2506.5864, found: 2506.2251 [M^+^].

#### 4.3.5 Compound Tz-Fl-BDP

Compound 7 (120 mg, 0.047 mmol) was dissolved in anhydrous dichloromethane (3 mL). Then DDQ (48 mg, 0.141 mmol) was added to the reaction mixture at 0 °C and stirred at room temperature for 1 h. After the complete oxidation of compound 7, which was monitored by TLC, triethylamine (0.09 mL, 0.705 mmol) was added. The reaction flask was cooled to 0 °C, and BF_3_. OEt_2_ (0.18 mL, 1.41 mmol) was added dropwise to the reaction mixture and stirred at room temperature for 1 h. Then, water was added to the reaction mixture and extracted thrice with dichloromethane. The combined organic layer was dried over anhydrous Na_2_SO_4_ and concentrated in a rotary evaporator. After evaporation of the solvent, the crude product was purified by silica gel column chromatography using neutral silica gel in 35% ethyl acetate in hexane as eluent to obtain 45 mg of Tz-Fl-BDP as a red solid. Yield: 36%; Mp: 98 °C; ^1^H NMR (400 MHz, CDCl_3_): *δ* 7.91–7.84 (m, 12H),7.79 (s, 6H), 7.70–7.66 (m, 6H), 7.36 (d, *J* = 8.0 Hz, 6H), 4.46(q, *J* = 7.2 Hz, 12H, –CH_2ester_), 2.09–2.05 (m, 12H), 2.01 (s, 18H, –CH_3_pyrrole), 1.44 (m, 36H), 1.19–1.05 (m, 60H), 0.79 (t, *J* = 6.8 Hz, 18H), 0.65 (m, 12H); ^13^C NMR (100 MHz, CDCl_3_): *δ* 169.51, 162.22, 152.64, 151.50, 148.10, 145.21, 144.30, 142.60, 142.03, 140.00, 139.50, 133.57, 133.21, 131.90, 129.44, 128.13, 128.07, 127.49, 126.26, 122.40, 121.29, 120.95, 119.75, 61.96, 55.60, 40.21, 31.71, 29.89, 29.15, 23.76, 22.55, 13.99, 12.54, 9.53; ^11^B NMR (128 MHz, CDCl_3_, proton decoupled): *δ* −0.60 (t, *J* = 28.03 Hz);^19^ F NMR (376 MHz, CDCl_3_): *δ* −141.07, −141.16, −141.22, −141.29. IR (KBr): 2924, 2846, 1641, 1594, 1378, 1247, 1177, 1108, 1014 cm^−1^. HRMS(*m*/*z*): calculated for C_165_H_198_B_3_F_6_N_9_O_12_: 2644.5343, found: 2644.6131 [M^+^].

## Conflicts of interest

The authors declare no competing financial interests.

## Supplementary Material

RA-016-D6RA05235E-s001

RA-016-D6RA05235E-s002

## Data Availability

The data supporting this article have been included as part of the supplementary information (SI). Supplementary information: ^1^H, ^13^C, ^11^B, ^19^F, IR, and mass spectra of all new compounds. Optimized structures in xyz format. See DOI: https://doi.org/10.1039/d6ra05235e.
