# Supplementary material for: A star-shaped triazine-BODIPY trimer with fluorene spacers: synthesis, aggregation, and environment-dependent photophysical properties
Source: RSC Adv. 2026 Sep 21;16(44):56141–54. doi: 10.1039/d6ra05235e (PMC13592504; doi:10.1039/d6ra05235e)
Supplement: Supplementary file 1 [file RA-016-D6RA05235E-s002.pdf]

Supplementary Information (SI) for RSC Advances.
This journal is © The Royal Society of Chemistry 2026

Star-Shaped Triazine–BODIPY Trimer with Fluorene Spacer: Synthesis, Aggregation, and Environment-Dependent Photophysical Properties

Laxmipriya Nayak[a], Harshal V Barkale[b], Subhadeep Acharya[a], Supriya Routray[a], Simran Pattnaik[a], Nilanjan Dey[b,*], Rashmirekha Satapathy[a,*]

[a] Department of Chemistry, Ravenshaw University, Cuttack-753003, Odisha, India
[b] Deprtment of Chemistry, Birla Institute of Technology and Science, Pilani, Hyderabad-500078, Telangana, India.

[*]Corresponding Author
Email: nilanjan@hyderabad.bits-pilani.ac.in; rashmi@ravenshawuniversity.ac.in

Contents **Page No.**

NMR Spectra…………………………………………………………………. 2

Mass Spectra………...............……………………………………………………… 8

Computational Analysis.. 11

Photophysical Studies.. 12

NMR spectra

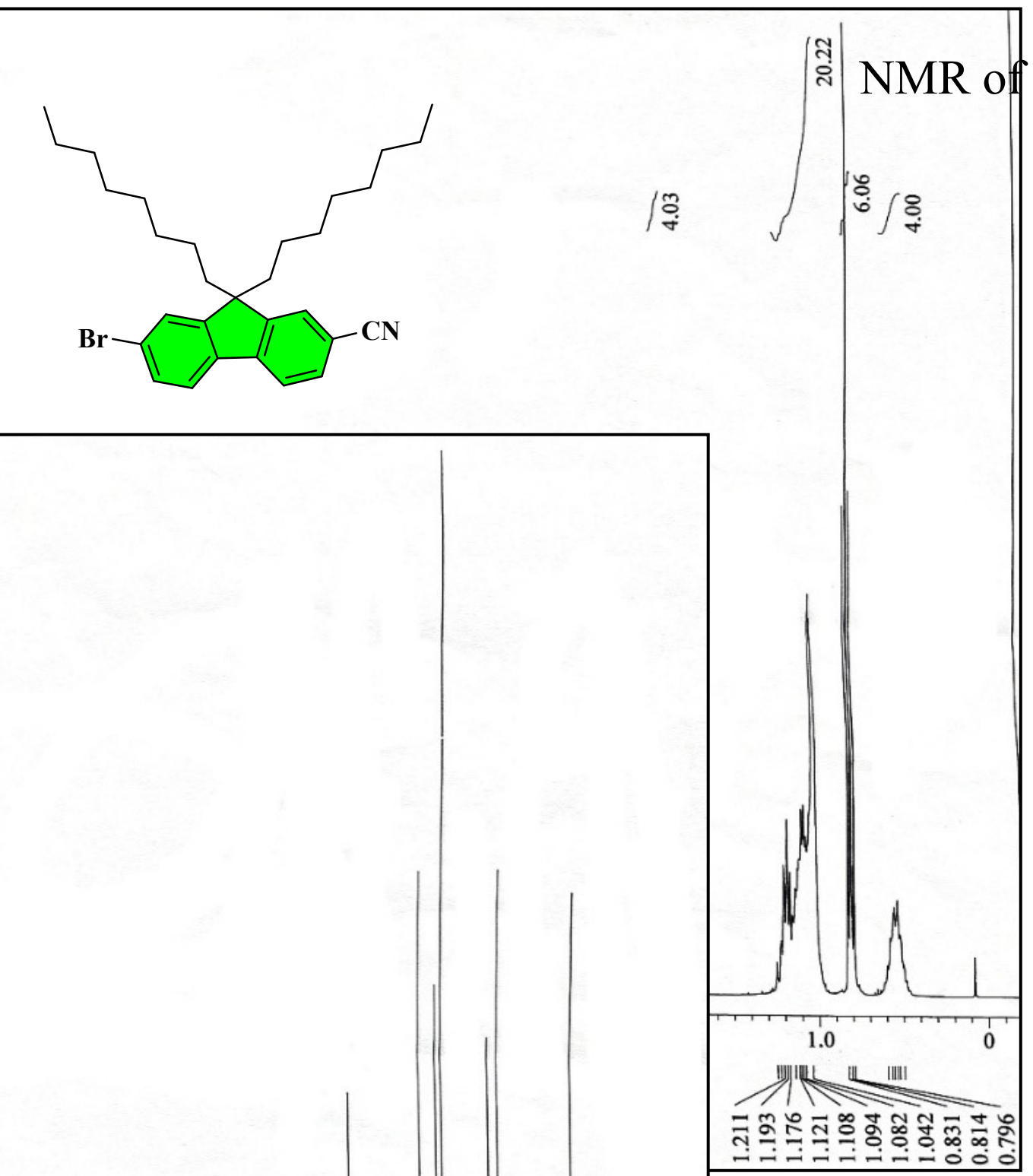

Figure S1: ^{1}H NMR of compound **2**

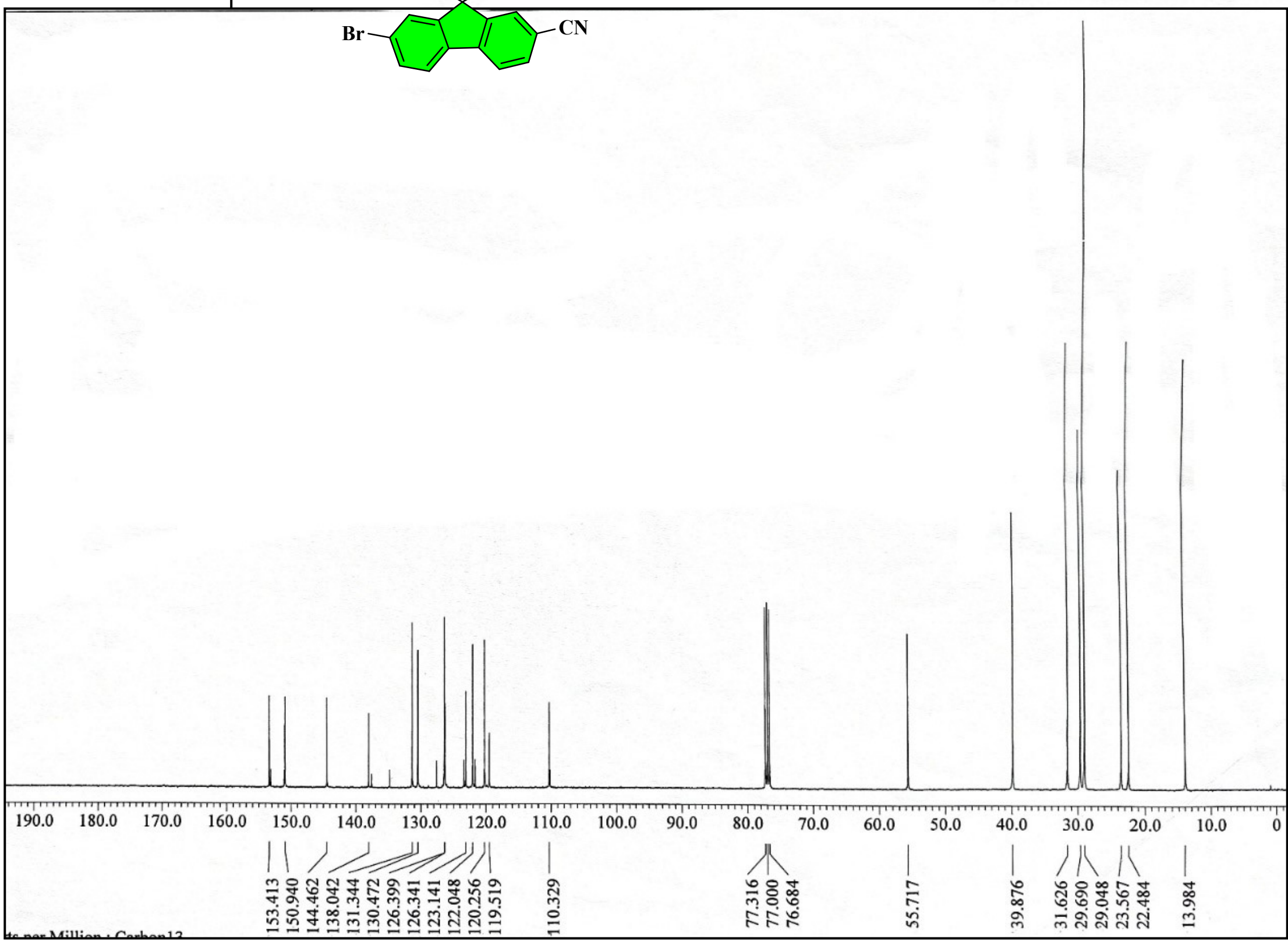

Figure S2: ^{13}C NMR of compound **2**

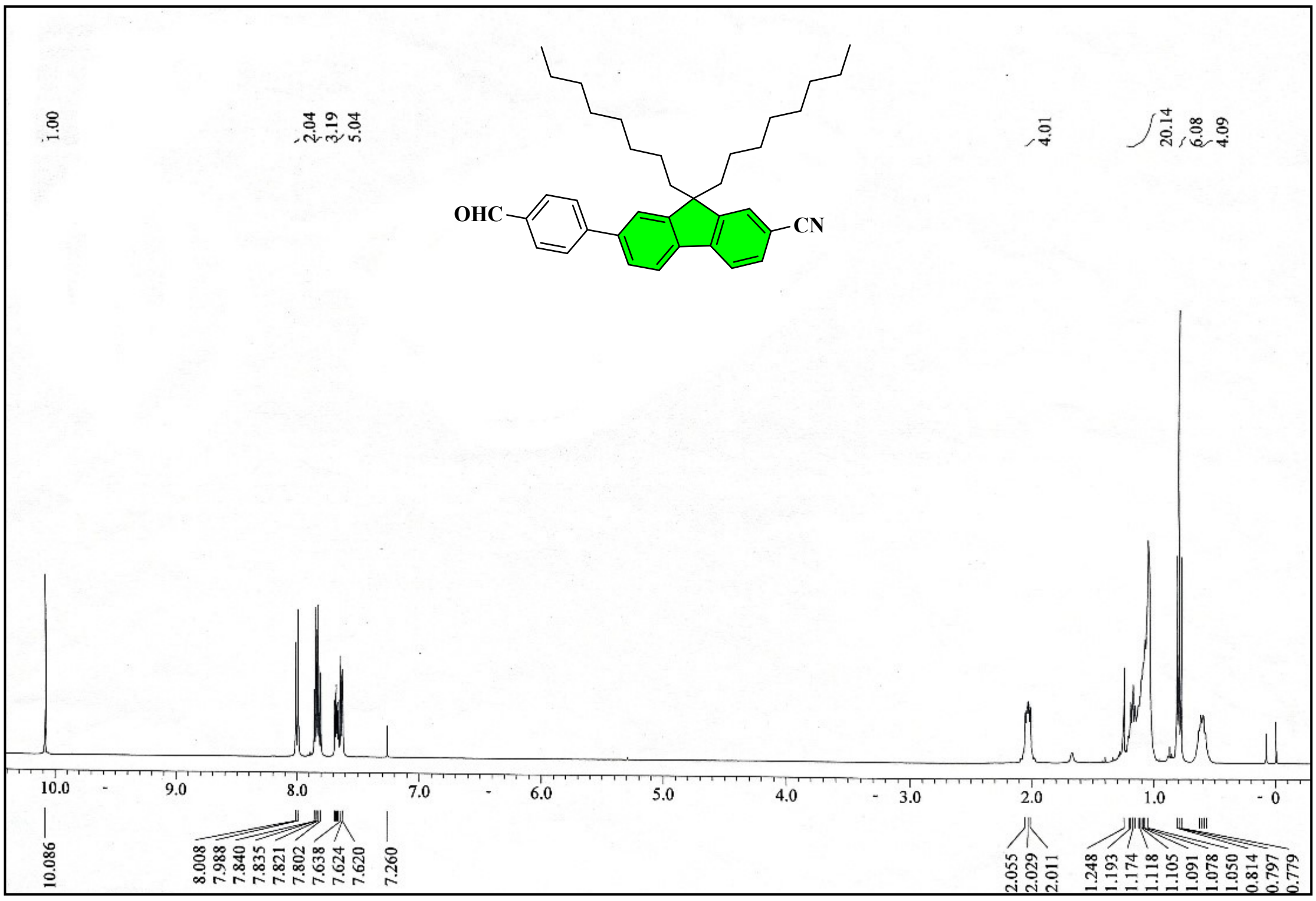

Figure S3: ^{1}H NMR of compound **4**

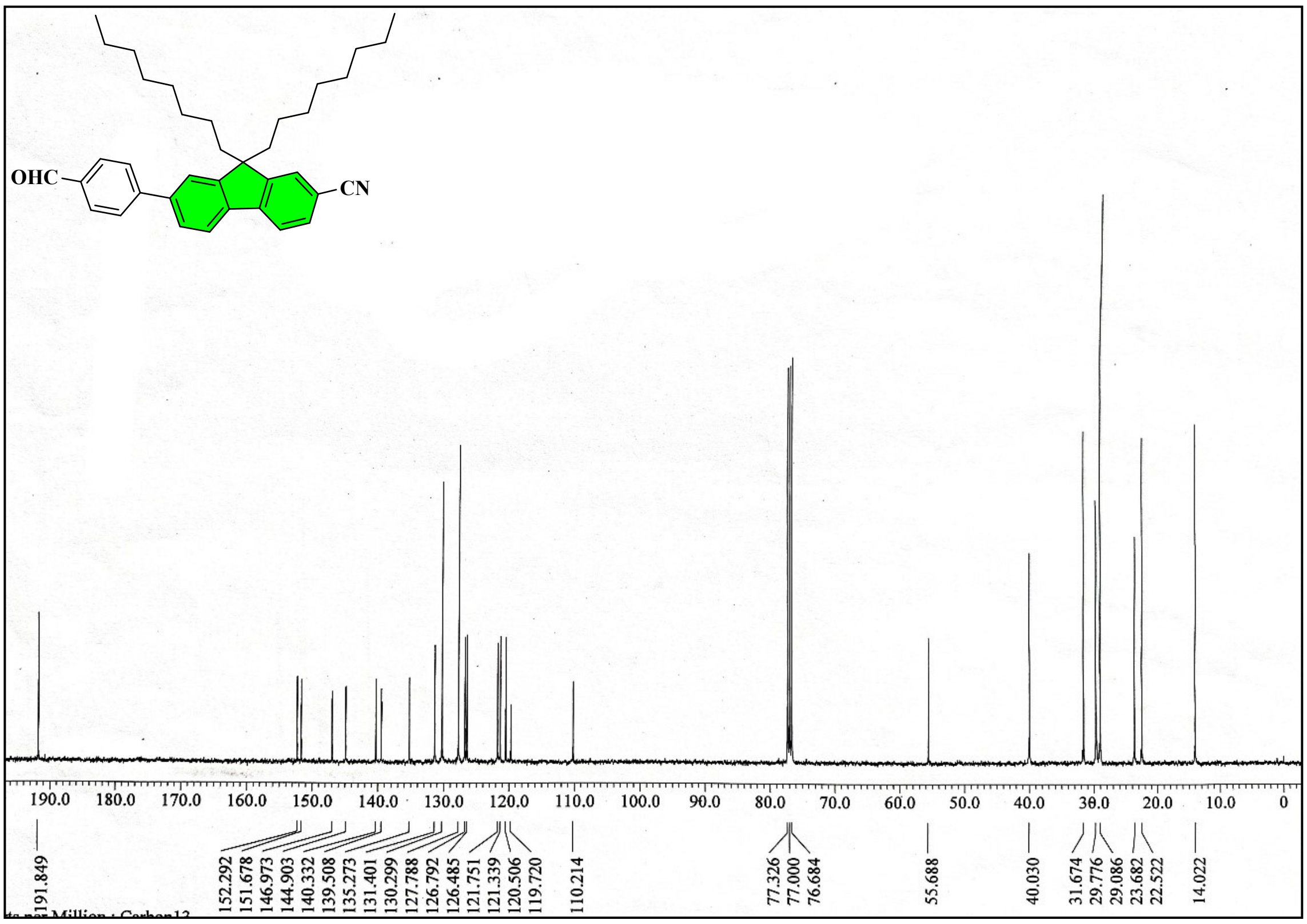

Figure S4: ^{13}C NMR of compound **4**

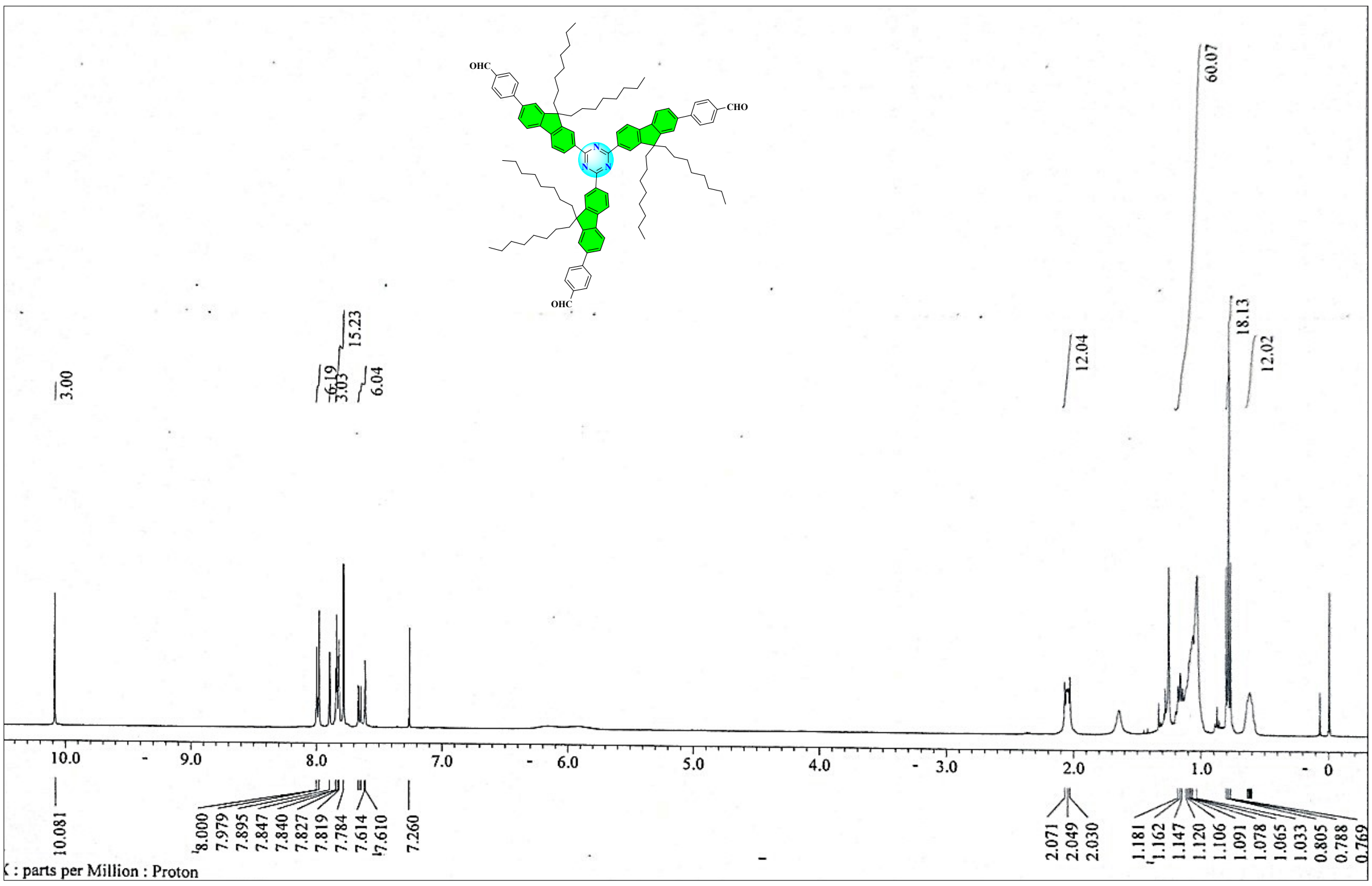

Figure S5: ^{1}H NMR of compound **5**

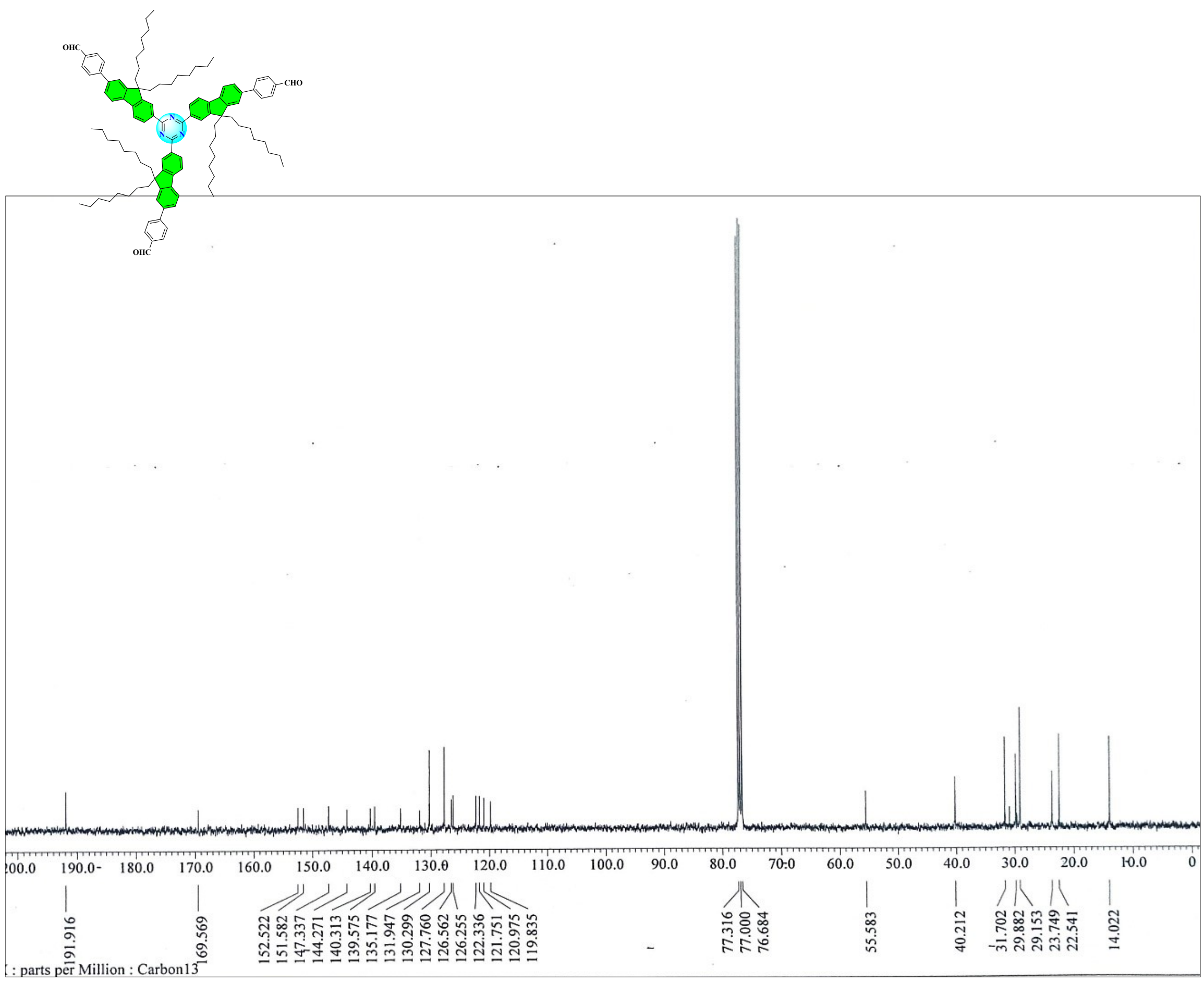

Figure S6: ^{13}C NMR of compound **5**

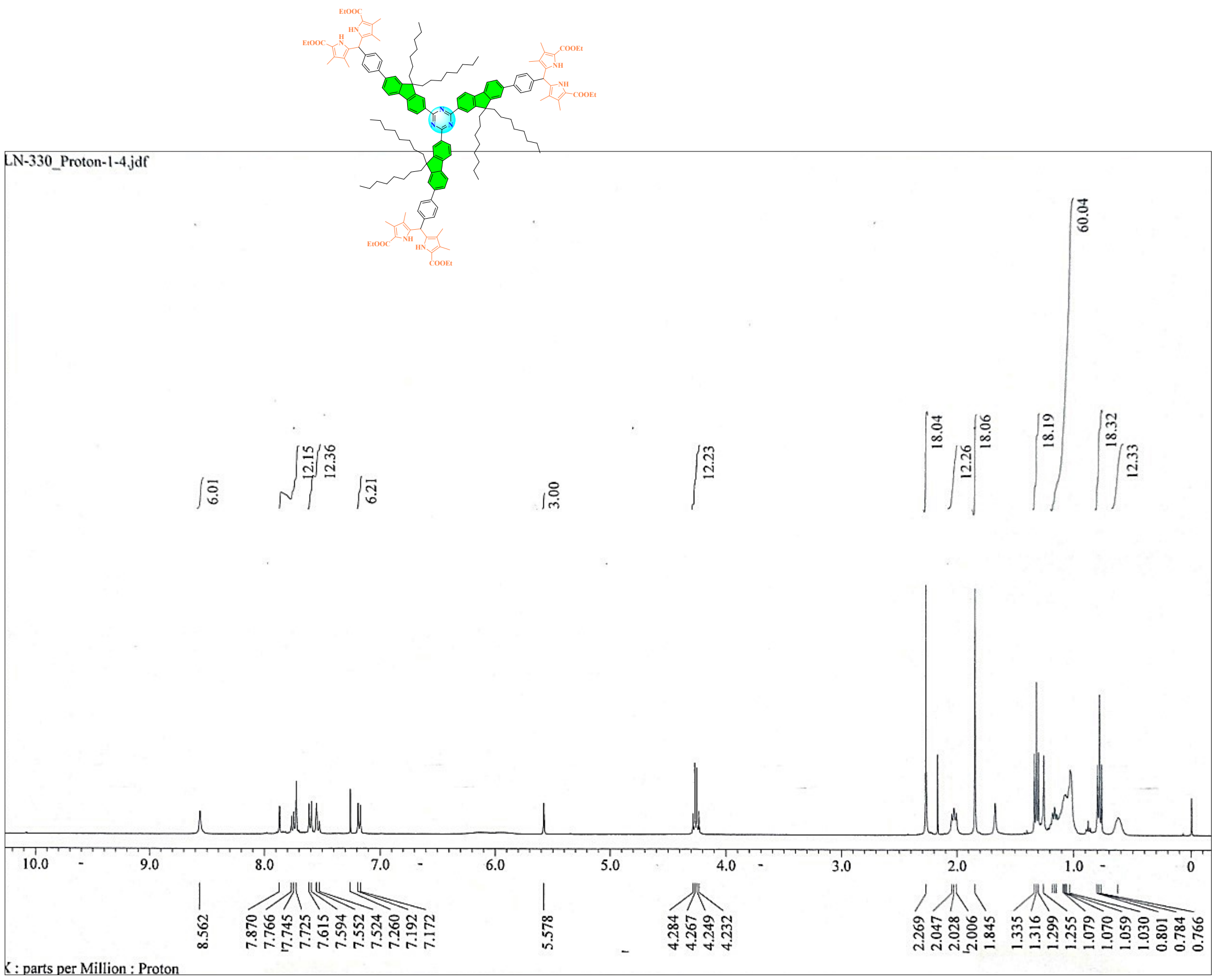

Figure S7: ^{1}H NMR of compound **7**

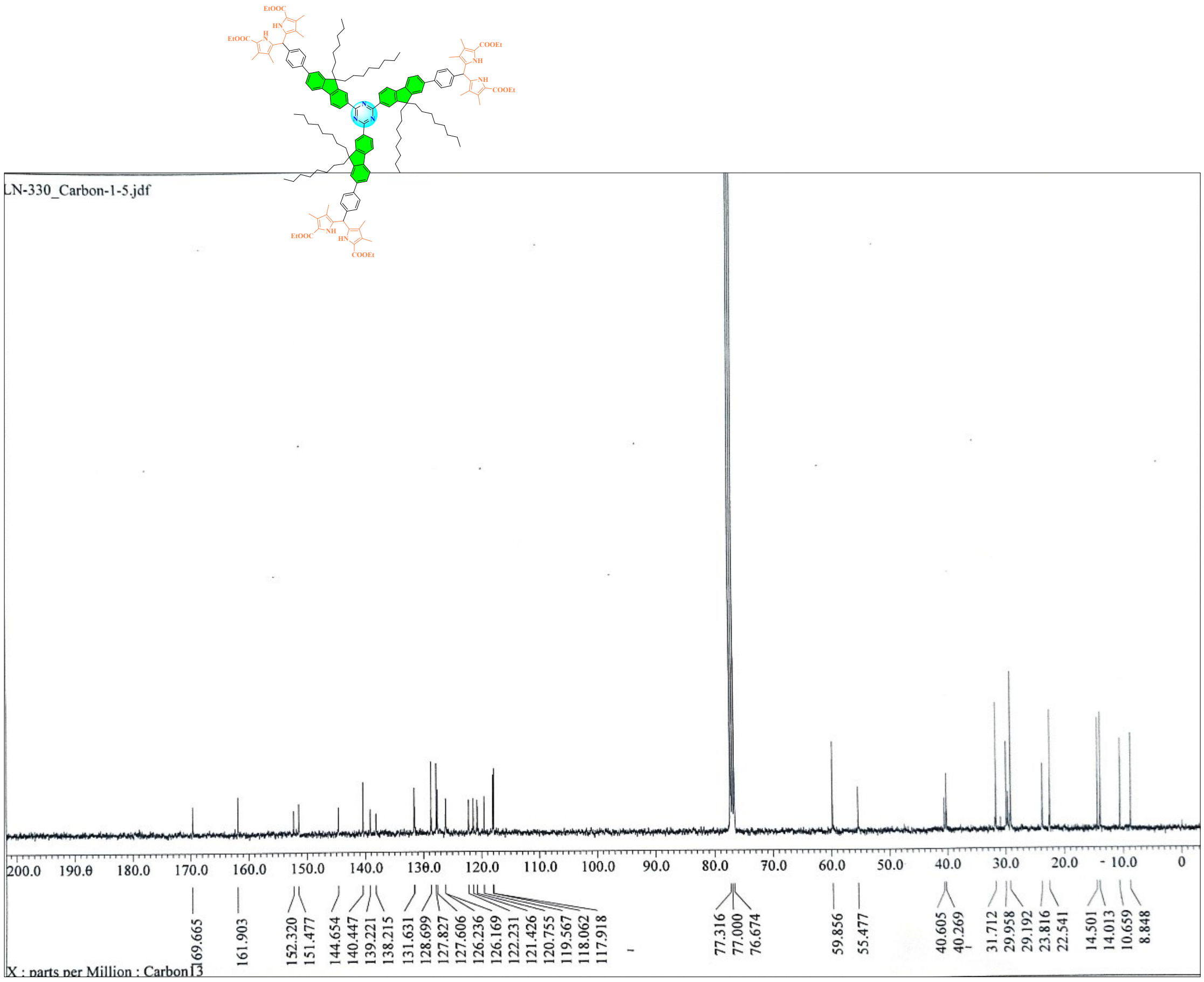

Figure S8: ^{13}C NMR of compound **7**

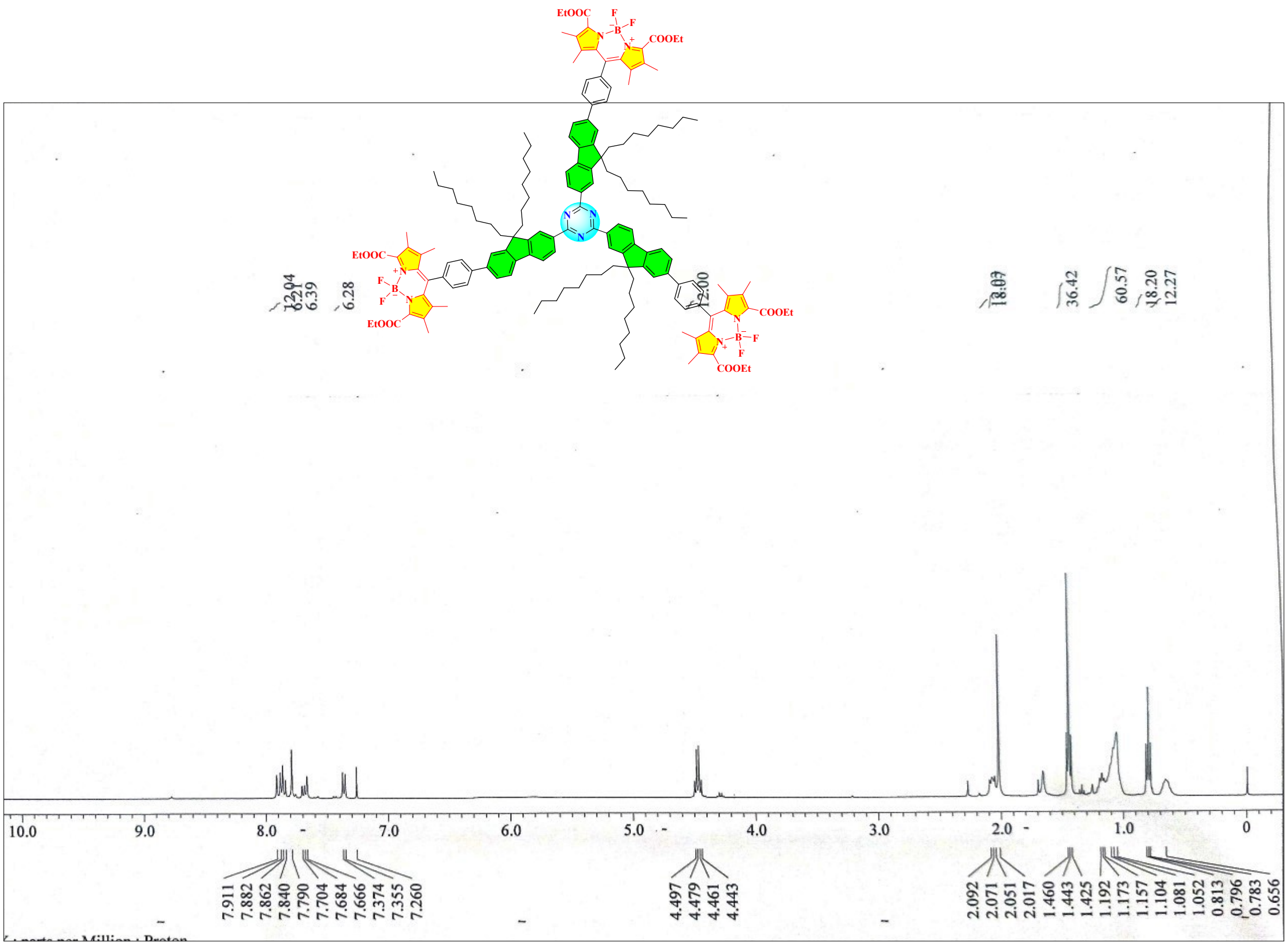

Figure S9: ^{1}H NMR of **TZ-Fl-BDP**

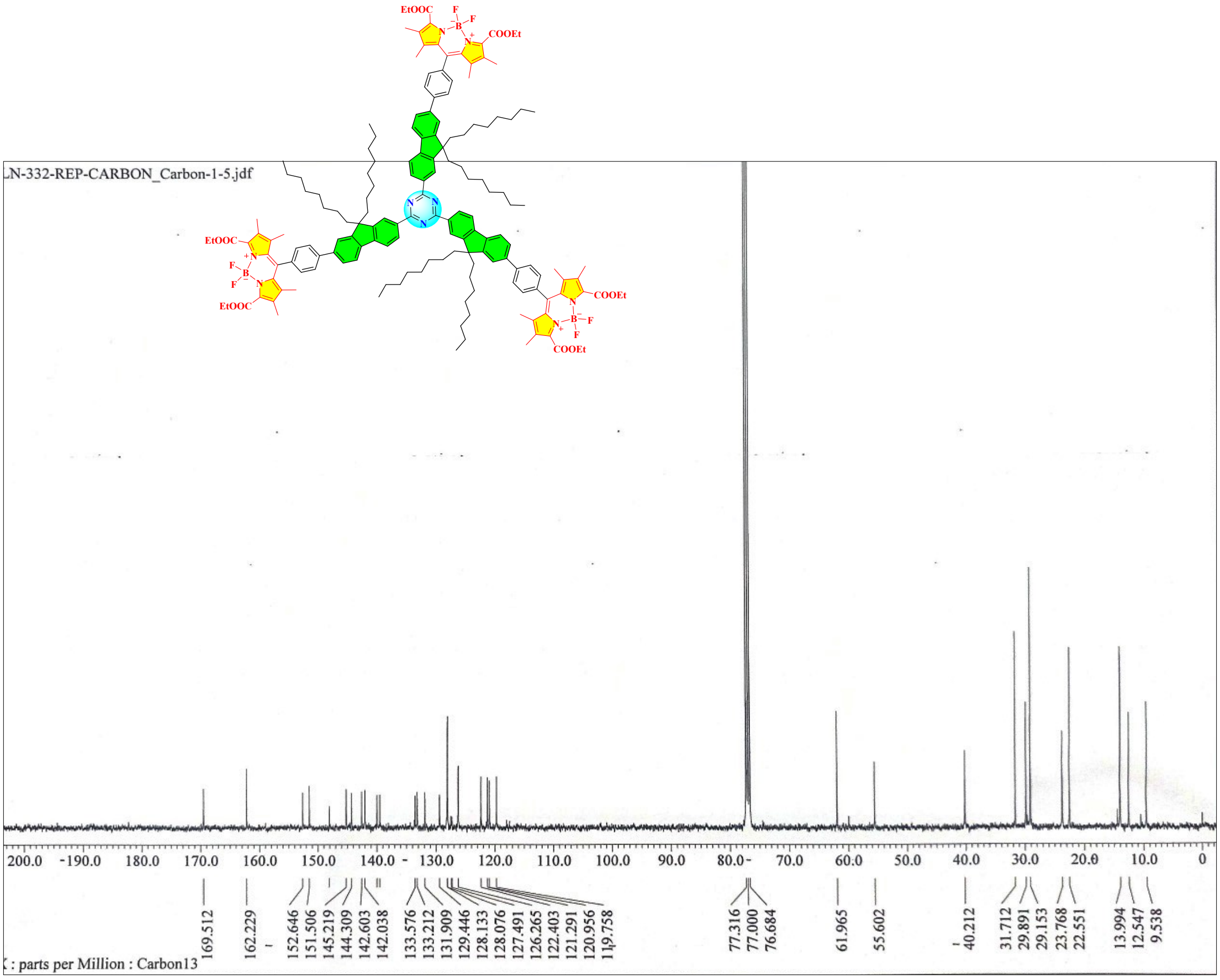

Figure S10: ^{13}C NMR of **TZ-Fl-BDP**

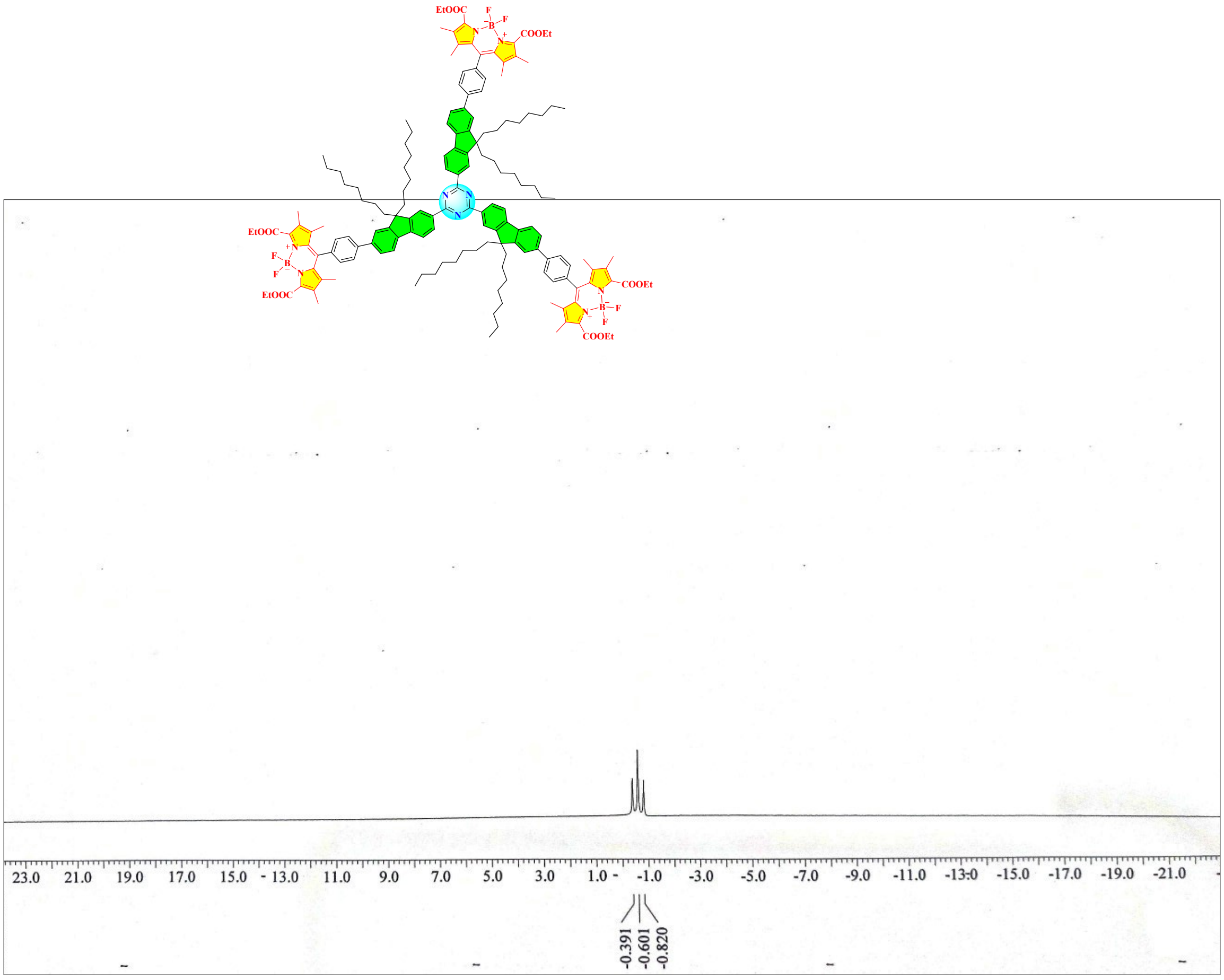

Figure S11: ^{11}B NMR of **TZ-Fl-BDP**

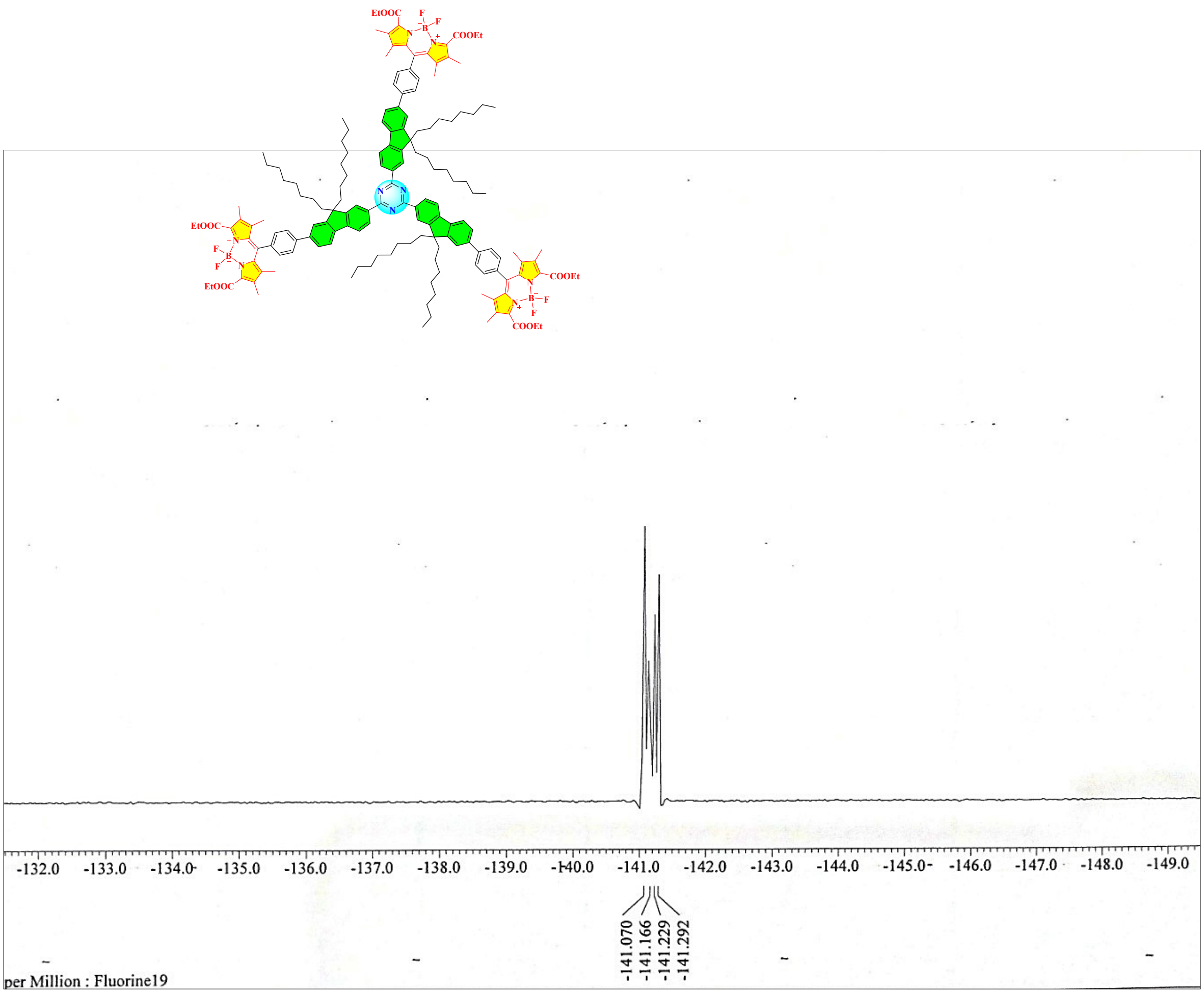

Figure S12: ^{19}F NMR of **TZ-Fl-BDP**

<u>Mass spectra</u>

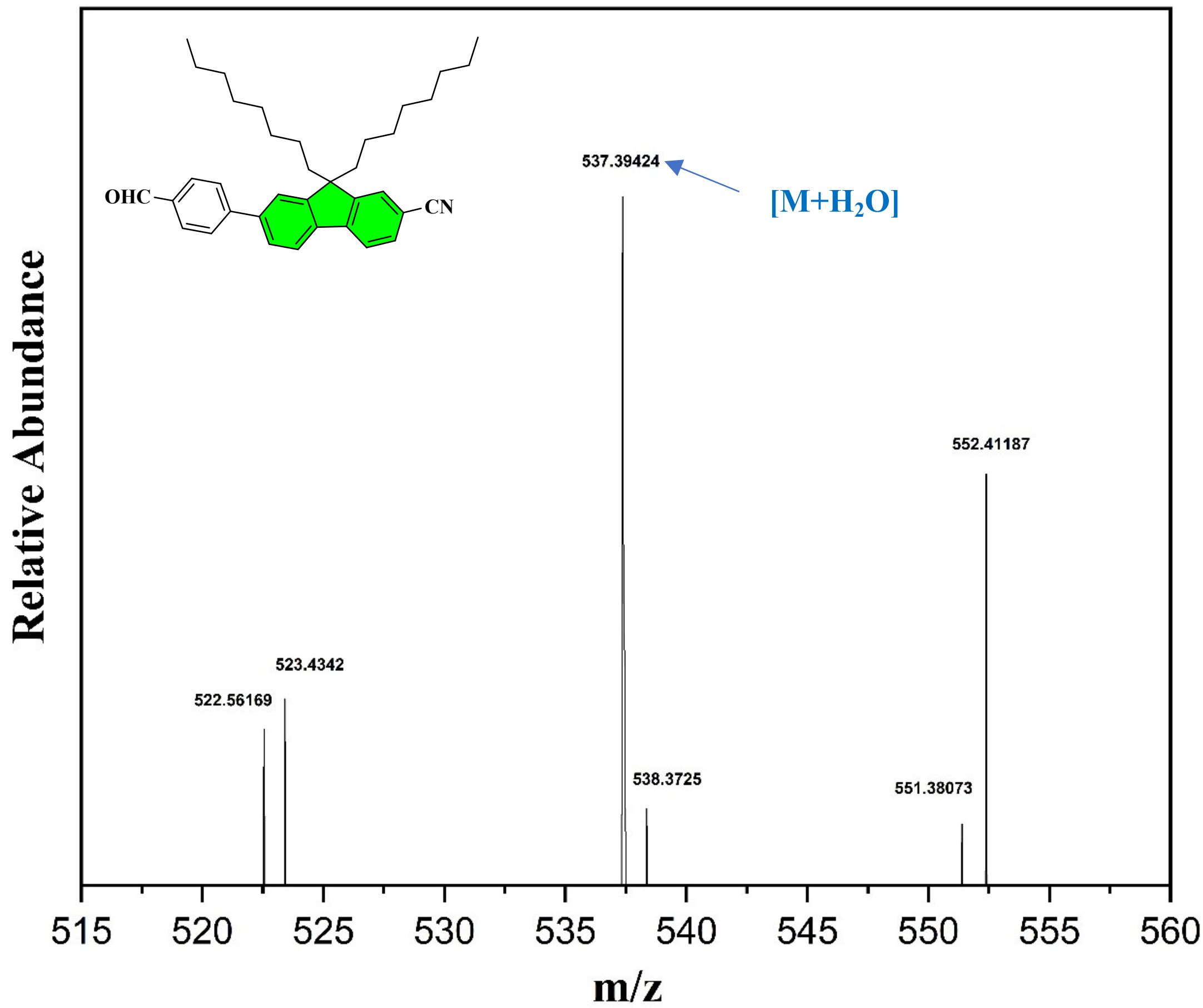

Figure S13: Mass spectra of **Compound 4**

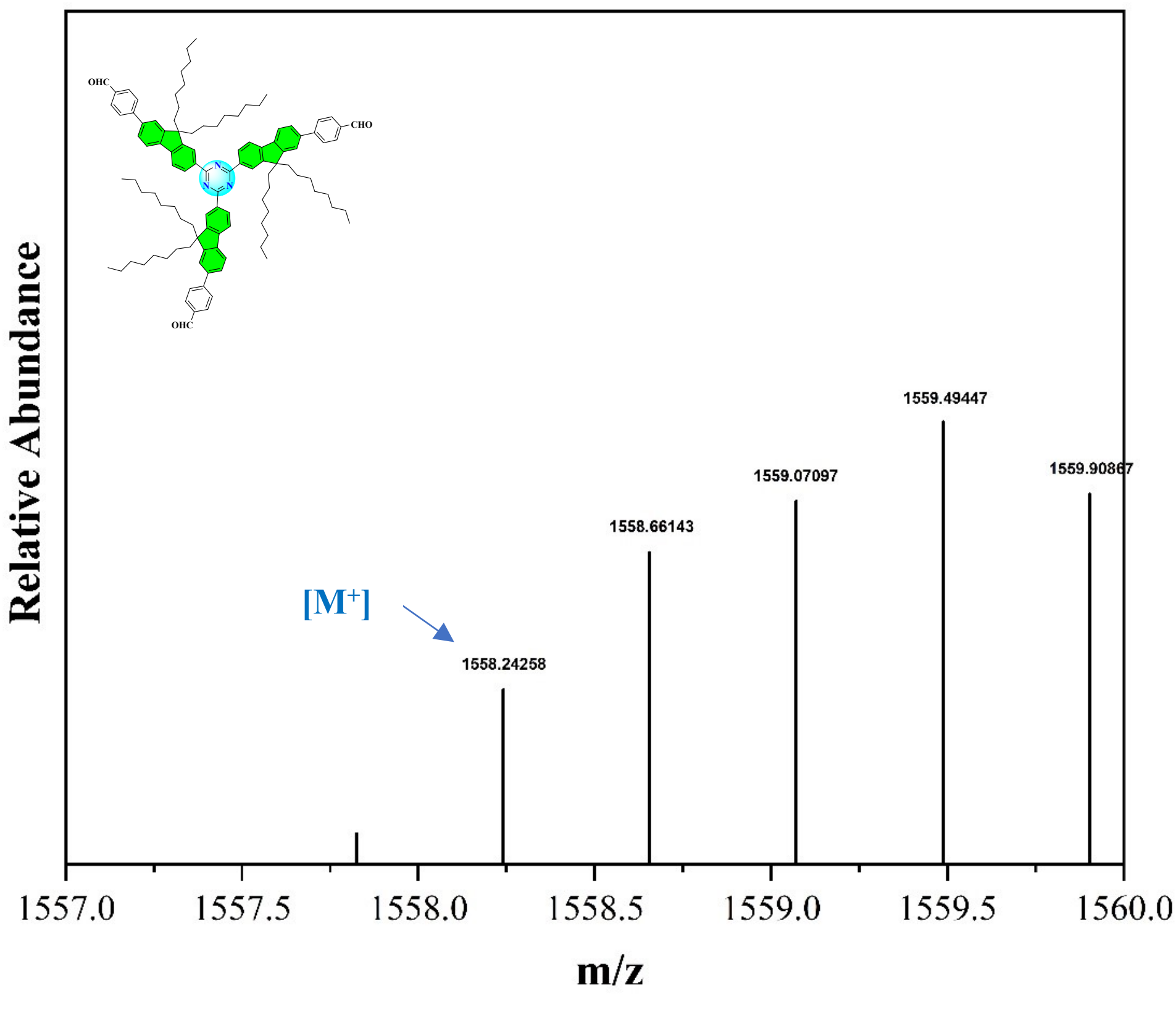

Figure S14: Mass spectra of **Compound 5**

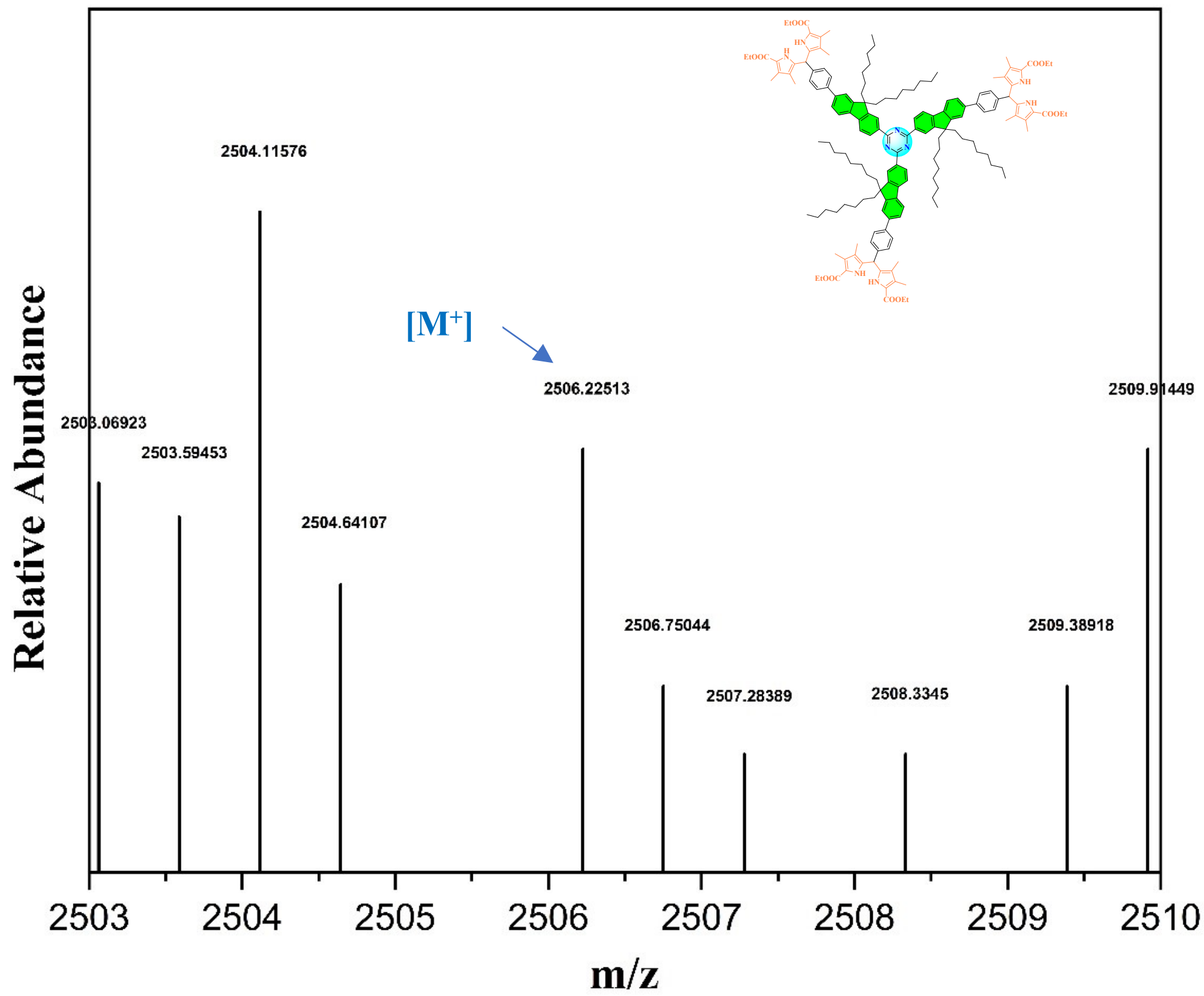

Figure S15: Mass spectra of **Compound 7**

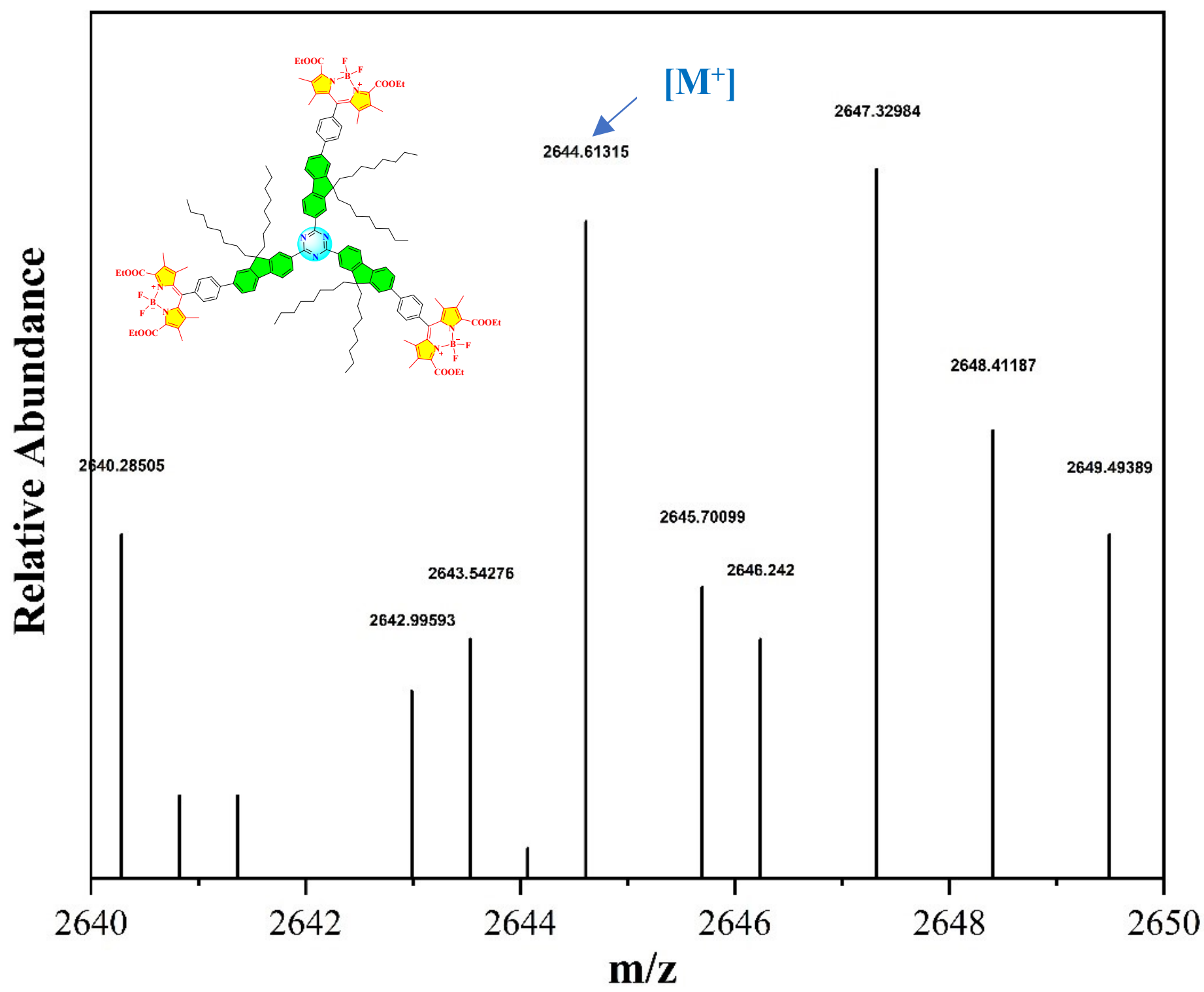

Figure S16: Mass spectra of **Tz-Fl-BDP**

Computational Analysis

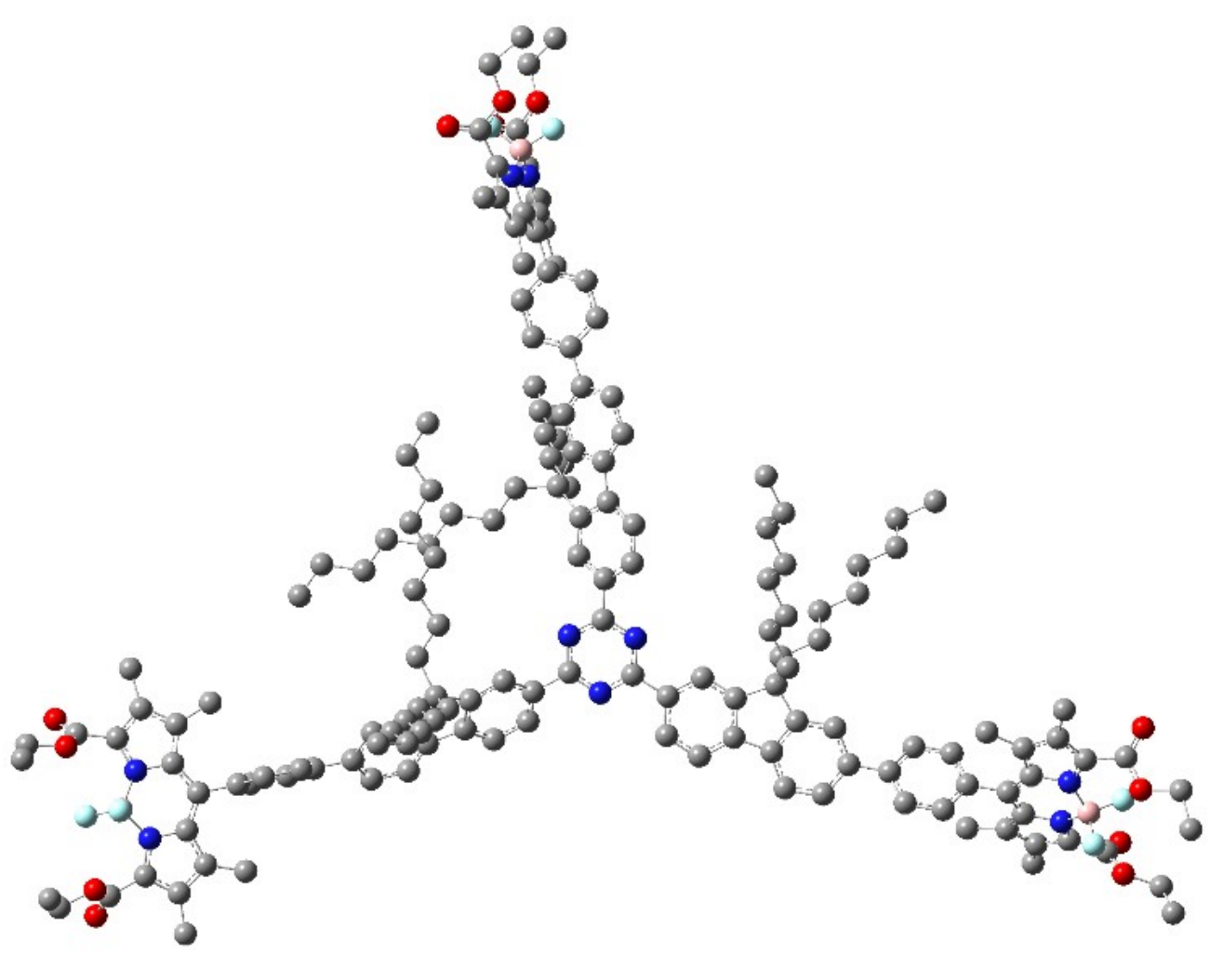

Figure S17: Optimized geometries of **Tz-Fl-BDP** (hydrogen atoms are removed for clarity)**.**

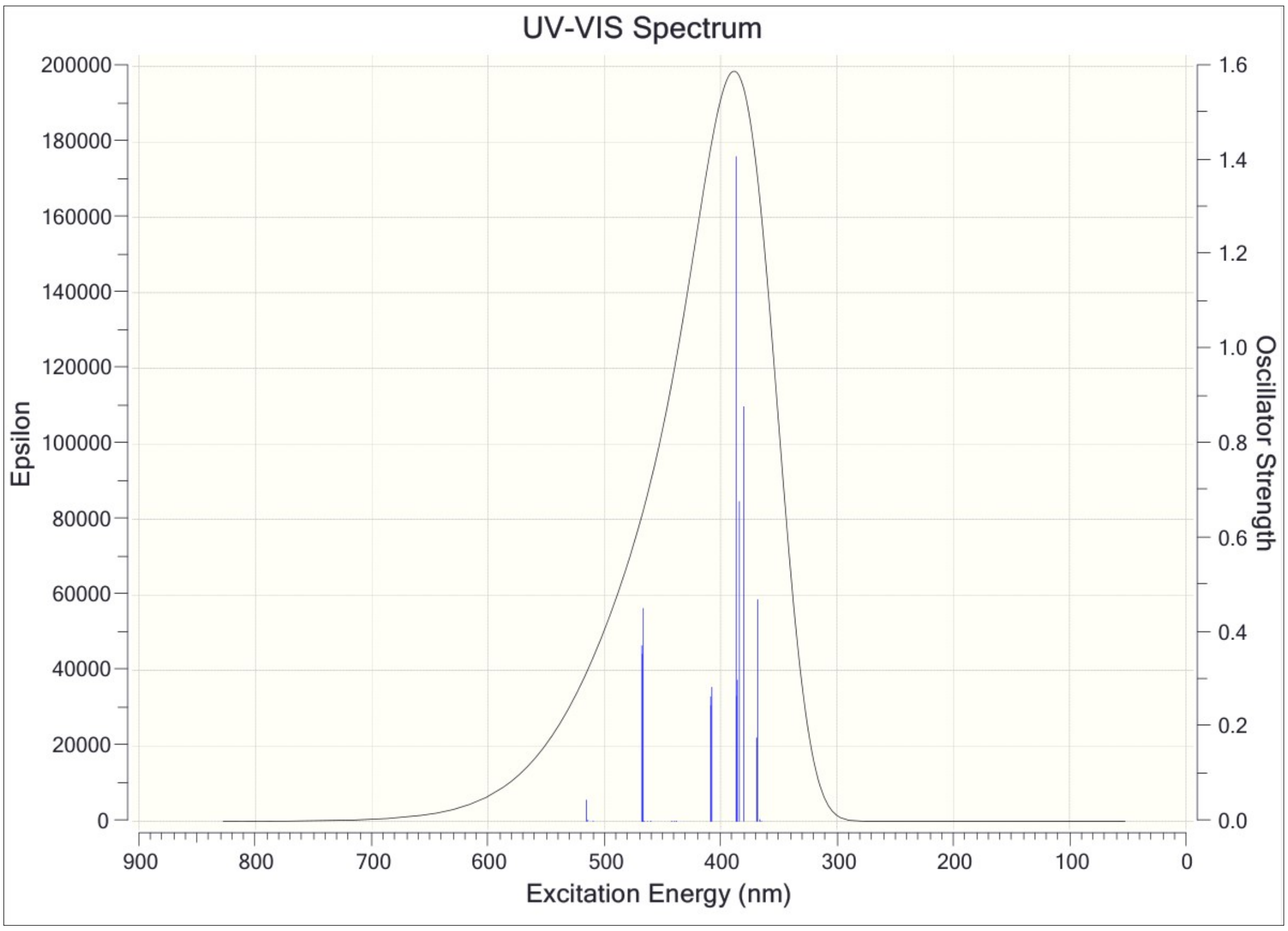

Figure S18: TD-DFT plot of **Tz-Fl-BDP** calculated at B3LYP functional with the 6-311++G(d,p) basis set, incorporating solvent effects through the polarizable continuum model (PCM) with dichloromethane (DCM) as the solvent.

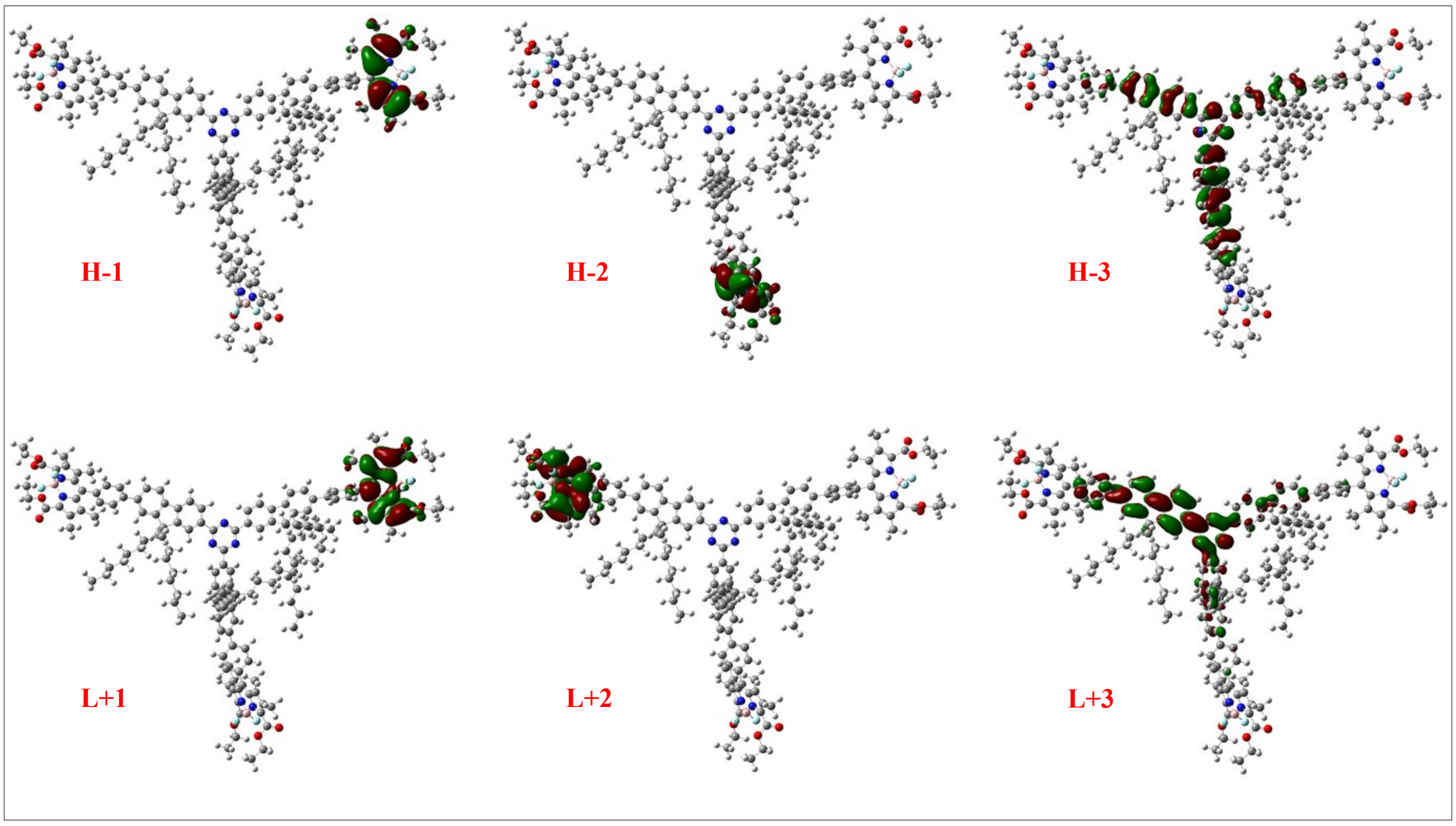

Figure S19: List of primary orbitals of **Tz-Fl-BDP** which are contribute to the calculated transitions.

Photophysical Studies

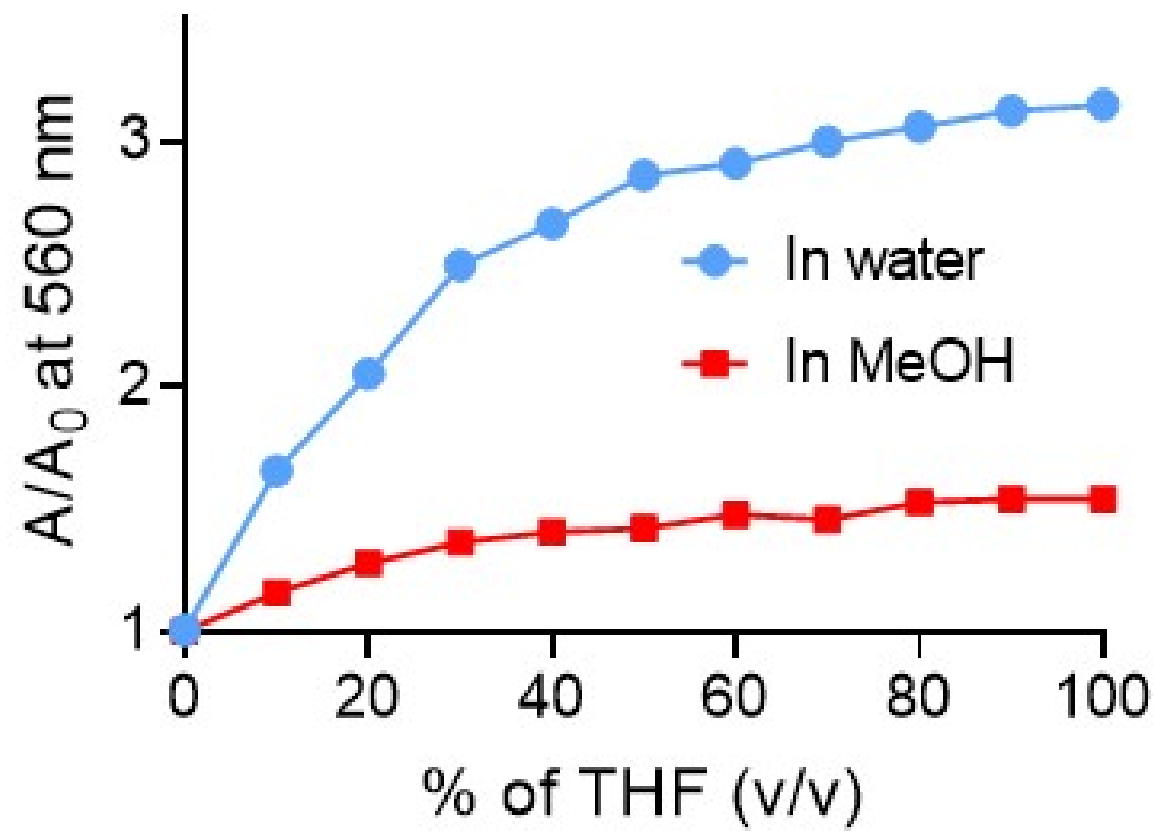

Figure S20: A/A_0 vs. % THF plot of **Tz-Fl-BDP**
